# Mechanism of pH-dependent activation of the sodium-proton antiporter NhaA

**DOI:** 10.1038/ncomms12940

**Published:** 2016-10-06

**Authors:** Yandong Huang, Wei Chen, David L. Dotson, Oliver Beckstein, Jana Shen

**Affiliations:** 1 Department of Pharmaceutical Sciences, University of Maryland School of Pharmacy, Baltimore, Maryland 21201, USA; 2 Department of Physics, Arizona State University, Tempe, Arizona 85287, USA; 3 Center for Biological Physics, Arizona State University, Tempe, Arizona 85287, USA

## Abstract

*Escherichia coli* NhaA is a prototype sodium-proton antiporter, which has been extensively characterized by X-ray crystallography, biochemical and biophysical experiments. However, the identities of proton carriers and details of pH-regulated mechanism remain controversial. Here we report constant pH molecular dynamics data, which reveal that NhaA activation involves a net charge switch of a pH sensor at the entrance of the cytoplasmic funnel and opening of a hydrophobic gate at the end of the funnel. The latter is triggered by charging of Asp164, the first proton carrier. The second proton carrier Lys300 forms a salt bridge with Asp163 in the inactive state, and releases a proton when a sodium ion binds Asp163. These data reconcile current models and illustrate the power of state-of-the-art molecular dynamics simulations in providing atomic details of proton-coupled transport across membrane which is challenging to elucidate by experimental techniques.

## Abstract (web summary)

The pH dependence of the activity of *Escherichia coli* main sodium-proton antiporter NhaA is still not fully understood. Here, the authors use continuous constant pH molecular dynamics simulations to identify NhaA proton carrier residues and elucidate its gating and ion transport processes.

## Graphical abstract

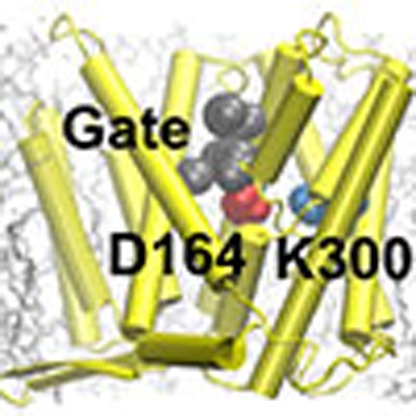

Sodium-proton antiporters play a major role in regulating H^+^ and Na^+^ concentrations in the cell. NhaA from *Escherichia coli* is perhaps the most widely studied one, as evident from two high-resolution crystal structures and abundant biochemical and biophysical data^1^. NhaA exchanges two protons in the periplasm for one sodium in the cytoplasm, and its activity is tightly regulated by pH (ref. 2). Below pH 6.5 NhaA appears inactive, while between pH 7 and 8.5 the sodium efflux rate increases dramatically, with a maximum around pH 8.7 (refs 3, 4, 5). The first atomic-resolution crystal structure of NhaA solved at pH 4 (ref. 6) revealed a bundle of 12 transmembrane helices (TMs) forming the inward-facing conformation where the ion passage funnel opens to the cytoplasm (Fig. 1a). Remarkably, TMs IV and XI are interrupted by extended chains crossing each other in the middle of the membrane, where Asp163, Asp164, Asp133, Thr132 and Lys300 form the active site responsible for ion binding^1^. Asp163 and Asp164 are highly conserved and their mutations completely abolish the activity of NhaA^7^,^8^,^9^. Thr132, Asp133 and Lys300 are also well conserved and their mutations impair the antiporter activity^9^,^10^,^11^. Biophysical and biochemical experiments suggested that Asp163, Asp164 and Thr132 are involved in ion binding^11^.

Based on the first crystal structure of NhaA^6^, Poisson-Boltzmann (PB) continuum electrostatics calculations with an effective protein dielectric constant of 4 showed that the p*K*_a_'s of Asp163 and Asp164 are shifted above 15, thus suggesting the two residues as the proton carriers required for exchanging a sodium ion^12^,^13^. Supported by Cys-scanning mutagenesis experiments, Padan and coworkers proposed that a ‘pH sensor', a cluster of residues at the entrance of the cytoplasmic funnel (Fig. 1a), is responsible for the pH dependence of the NhaA activity^14^,^15^,^16^. They further proposed that a pH signal from the cytoplasm results in deprotonation of a single pH-sensing residue, presumably Glu78 based on the PB calculations^12^, which is transduced down to the active site triggering a conformational change and consequently the p*K*_a_ downshifts of Asp163 and Asp164, leading to the release of two protons^1^,^17^.

The above pH activation-based allosteric mechanism^1^ was recently challenged by a new set of data. Electrophysiology experiments showed that, under symmetric, saturating sodium concentration and pH 8.5 on the periplasmic side, reverse transport persists when pH on the cytoplamic side is lowered from 8 to 5 (ref. 4). Together with studies on related antiporters^18^, it prompted the proposal of an alternative model, in which Na^+^ and H^+^ directly compete for binding to a common site^19^. The identity of the residues involved in the binding site remained however speculative. Measurements of lithium binding to mutants of Lys300 suggested its positive charge and p*K*_a_ are essential for antiporter activity, possibly by affecting sodium binding^11^. Additional evidence for the involvement of Lys300 came from a new crystal structure of NhaA^20^, solved at the same pH condition and diffraction resolution as the previous one^6^, showing a salt bridge between Lys300 and Asp163 (Fig. 1b). An equivalent salt bridge is also seen in the crystal structures (solved at pH 7.8) of the homologous sodium-proton antiporter NapA from *Thermus thermophilus* in the outward-21 and inward-facing states^22^. Like NhaA, NapA is electrogenic and therefore the Lys300–Asp163 salt bridge is likely of functional importance.

The competitive binding model is also supported by the recent molecular dynamics (MD) simulations based on the new crystal structure of NhaA^20^ and combinations of protonation states of Asp163/Asp164/Lys300 (ref. 20). The simulations revealed that when Asp164 was protonated, no sodium binding occurred, and the salt bridge remained intact between charged Asp163 and Lys300; however, when Asp164 was deprotonated, sodium entered and bound to Asp164 and Asp163, which destabilized the salt bridge^20^. p*K*_a_ calculations using PROPKA^23^ based on the trajectory snapshots suggested that Lys300 is likely charged when it is in salt bridge with Asp163 (ref. 20). These results, together with the recent crystal structures of NapA^21^,^22^ as well as biophysical and biochemical experiments^4^,^11^,^18^, led to a modified competitive binding mechanism^20^. At low pH in the inward-facing state of NhaA, protons are bound to Asp164 and Lys300; the latter forms a salt bridge with the deprotonated Asp163. Sodium entrance to the active site results in binding to Asp163 and Asp164. The former leads to the breakage of the salt bridge and release of a proton from Lys300, while the latter represents a direct competition with a proton and leads to the deprotonation of Asp164. Following the proton-sodium exchange, a conformational switch from the inward- to the outward-facing state takes place. Thus, a major difference from the allosteric mechanism is whether the sodium-proton exchange requires a pH-induced activation step^1^. Most recently, based on the new crystal structure^20^, the function of NhaA was explored by Warshel and coworker using a novel approach which combines semi-macroscopic p*K*_a_ calculations with Monte-Carlo (MC) simulations^24^. This study supported Asp163 and Asp164 as the two proton carriers^12^ and the recent competitive binding^4^ and alternating access models^20^. Further, it was able to reproduce and rationalize features of the observed antiport activity, including the 2:1 stoichiometry and pH activity profile^4^.

Over the past decade, constant pH MD techniques have been developed that can offer atomic description of pH-coupled conformational dynamics (see refs 25, 26, 27, 28 and a recent review^29^). A promising alternative approach that can offer kinetic information has also been developed based on time-dependent MC simulations and the Empirical Valence Bond model^30^. Here we use the state-of-the-art continuous constant pH MD (CpHMD)^31^,^32^, which incorporates a membrane-embedded hybrid-solvent scheme and pH replica-exchange sampling protocol^33^, to explore the pH-dependent conformational dynamics of NhaA. Our study suggests that the pH sensor responds to pH, and Asp164 and Lys300 are the two proton carriers. Further, deprotonation of Asp164 allows opening of the cytoplasmic gate and binding of a sodium to the core residues including Asp163. The latter disrupts the Lys300–Asp163 salt bridge, leading to neutralization of Lys300 and a conformational change involving the region between the core and dimerization domains. Thus, our study offers an atomically detailed view of the pH activation and initial events of the sodium-proton exchange of NhaA, reconciling the current models regarding the intricate working of the antiporter.

## Results

### Overview of simulations

Three sets of pH replica-exchange CpHMD simulations of NhaA embedded in a POPC lipid bilayer were performed (Supplementary Table 1). The first set was initiated from the recent crystal structure^20^ (PDB ID: 4AU5), while the other two were initiated from the previous crystal structure^6^ (PDB ID: 1ZCD). During the simulations, all Asp, Glu, His and Lys sidechains were allowed to titrate. Unless otherwise noted, the discussion refers to the first set of simulations. The corresponding data from the other two sets are given in Supplementary Information and show general agreement with the first set of simulations. Convergence analyses are given in Supplementary Figs 1–4.

### pH sensor responds to the pH signal and attracts sodium

To explore the role of the ‘pH sensor', we examine the calculated p*K*_a_'s of ionizable residues located at the entrance of the cytoplasmic funnel of NhaA^15^, including Asp11 (TM I), Glu78, Arg81, and Glu82 (TM II), His243 (loop between TM VIII and TM IX), Lys249, Arg250, Glu252, His253 and His256 (TM IX) (Table 1). The previous continuum electrostatics calculation predicted Glu78 to have a p*K*_a_ in the physiological pH range^13^. Our simulations, however, showed that Glu78 and in fact, all acidic residues in the pH sensor have p*K*_a_ values below 4, which is not surprising, since they are surrounded by positively charged residues. All these acidic residues are thus fully deprotonated at the lowest pH conditions when activity can be experimentally detected, namely pH 6.5 (ref. 3) and more recently pH 5 (ref. 4). Thus, our simulations do not support Glu78 as the single pH-sensing residue. Rather, in light of the fact that mutations of all of the aforementioned pH sensor residues have been shown to significantly alter the pH profile of NhaA^14^,^16^, we reasoned that their collective charge helps attract sodium ions into the funnel above an activation pH. To test this hypothesis, we calculated the total net charge of the pH sensor residues as a function of pH. Remarkably, as pH increases from 3.5 to 9.5, the net charge decreases from 2.8 to −1.0, and the sign switch occurs around pH 7 (Fig. 2; Supplementary Fig. 5), close to the onset of measurable activity near pH 7 in biochemical^3^ and electrophysiology measurements^4^. Thus, we suggest that, rather than an individual residue, the pH sensor region collectively responds to the pH signal from the cytoplasm, recruiting sodium and activating NhaA as pH is increased to the active range, consistent with previous observations that negative potential on the vestibule of ion channels attracts cation into the pore^34^,^35^.

### Asp164 and Lys300 are the two proton carriers

To identify the proton carriers in the sodium-proton exchange process of NhaA, we turn to the calculated p*K*_a_'s of the core residues (Table 1). Asp164 is absolutely conserved in the sodium-proton antiporters across all species. Based on our simulations starting from both the recent and previous crystal structures, Asp164 is the only one among the three acidic core residues that has a p*K*_a_ (5.0/6.6/5.8), close to the activation pH for all three simulation runs. In contrast, the p*K*_a_ of Asp163 is below 4 in all three runs (2.4/3.5/3.9). Thus, Asp164 is the residue that can switch protonation state near the activation pH, in support of the hypothesis that Asp164 is one of the two proton carriers based on recent experiments^4^,^11^,^21^. Our data is also in agreement with the conclusions from the previous computational work^12^,^20^,^36^. Our simulations showed that, p*K*_a_(Asp164) >p*K*_a_(Asp133) >p*K*_a_(Asp163), consistent with the PROPKA calculation^23^ using the trajectories starting from the new crystal structure^20^. Asp164 has also been suggested as a proton carrier in the continuum electrostatics calculations based on the previous crystal structure^12^. This is not surprising, as our simulations initiated from the same crystal structure also assigned Asp164 with the highest p*K*_a_ among the three core acidic residues (Table 1). We note that our calculated p*K*_a_ of Asp163, which is downshifted from the model value, is in qualitative agreement with the p*K*_a_ obtained by the semi-macroscopic calculations considering neutral Asp164 and charged Lys300 (ref. 24; Supplementary Note 1).

**Figure 1:**
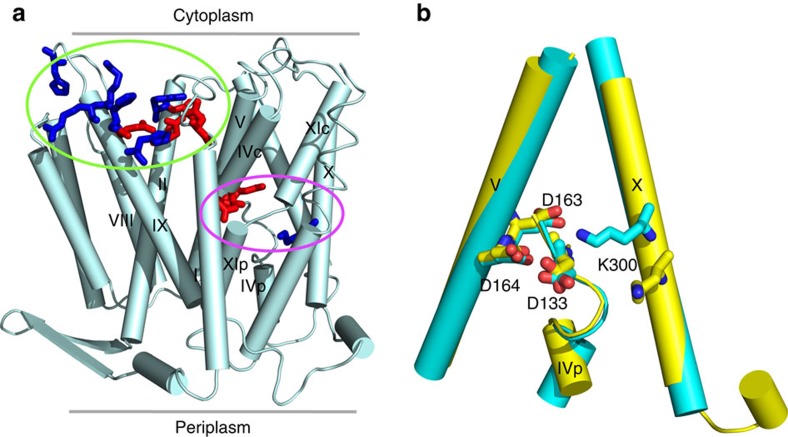
Structural features of NhaA. (**a**) The first (previous) atomic-resolution crystal structure of NhaA (pdb 1ZCD, resolution 3.45 Å)^6^ with residues in the pH sensor (entrance of the cytoplasmic funnel) and active site highlighted. The cytoplasmic funnel involves TMs IX, I, II and loops of TMs VIII and IX. The pH sensor contains Asp11 (TM I), Glu78, Arg81 and Glu82 (TM II), His243 (loop between TM VIII and IX), Lys249, Arg250, Glu252, His253 and His256 (TM IX). The active site contains Asp133 (between TMs IVc and IVp), Asp163 (TM V), Asp164 (TM V) and Lys300 (TM X). Acidic (Asp and Glu) and basic residues (Lys, Arg and His) are shown in red and blue, respectively. (**b**) Difference in the active site between the previous (yellow)^6^ and new crystal structures (cyan, pdb 4AU5, resolution 3.7 Å).

**Figure 2:**
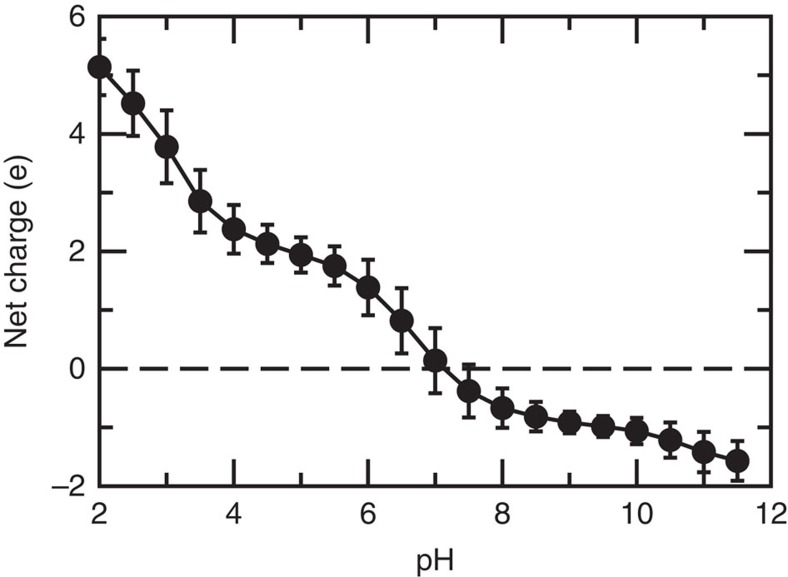
Net charge of the pH sensor switches sign at activation pH. Calculated total net charge of the residues in the pH sensor, Asp11, Glu78, Arg81, Glu82, His243, Lys249, Arg250, Glu252, His253 and His256. The error bars indicate the root-mean-squared fluctuations in the simulation. A horizontal line at net charge zero is drawn to guide the eye. Except for arginines which were kept in the charged state, all of the above residues were allowed to titrate in the simulation.

It is worthwhile noting the limitations of computational p*K*_a_ predictions. The PB-estimated p*K*_a_ of Asp164 (about 13)^12^ is too high, consistent with the benchmark study of PB-based p*K*_a_ predictions of interior residues using the same effective protein dielectric constant (*ɛ*_p_=4)^37^. Although overestimation of p*K*_a_ shifts can be dampened by adopting a larger *ɛ*_p_ to implicitly account for reorganization of the interior polar groups and water penetration, the improvement is limited^37^, as a single dielectric constant is insufficient to accurately capture both self energy and charge–charge interactions^38^. On the other hand, the CpHMD-estimated p*K*_a_ of Asp164 is likely too low (by up to 2 units^39^), as the underlying GBSW model^40^ underestimates desolvation^33^,^41^, due to the use of van der Waals surface which excludes solvent-inaccessible crevices from solute cavity, reflecting the inherent difficulty in treating solute–solvent dielectric transition by GB models^38^. However, the extent of the error is fortuitously cancelled by the overestimation of desolvation due to inadequate structural relaxation^33^,^41^. As a result, the calculated p*K*_a_'s of internal groups from the hybrid-solvent CpHMD simulations show surprisingly small deviations from experiment^33^,^39^. The second limitation is related to the incomplete sampling in CpHMD simulations, which manifests itself in the dependence of the calculated p*K*_a_'s on the initial configuration. As a result, the calculated p*K*_a_'s of Asp164 from the simulations initiated from the earlier crystal structure are 0.8/1.6 units higher than the p*K*_a_ from the simulation based on the recent crystal structure, because of the significant deviation between the two structures in this region (Fig. 1b). Given sufficient sampling (not within current computational resources), however, identical p*K*_a_'s should be obtained regardless of the starting structure.

**Figure 3:**
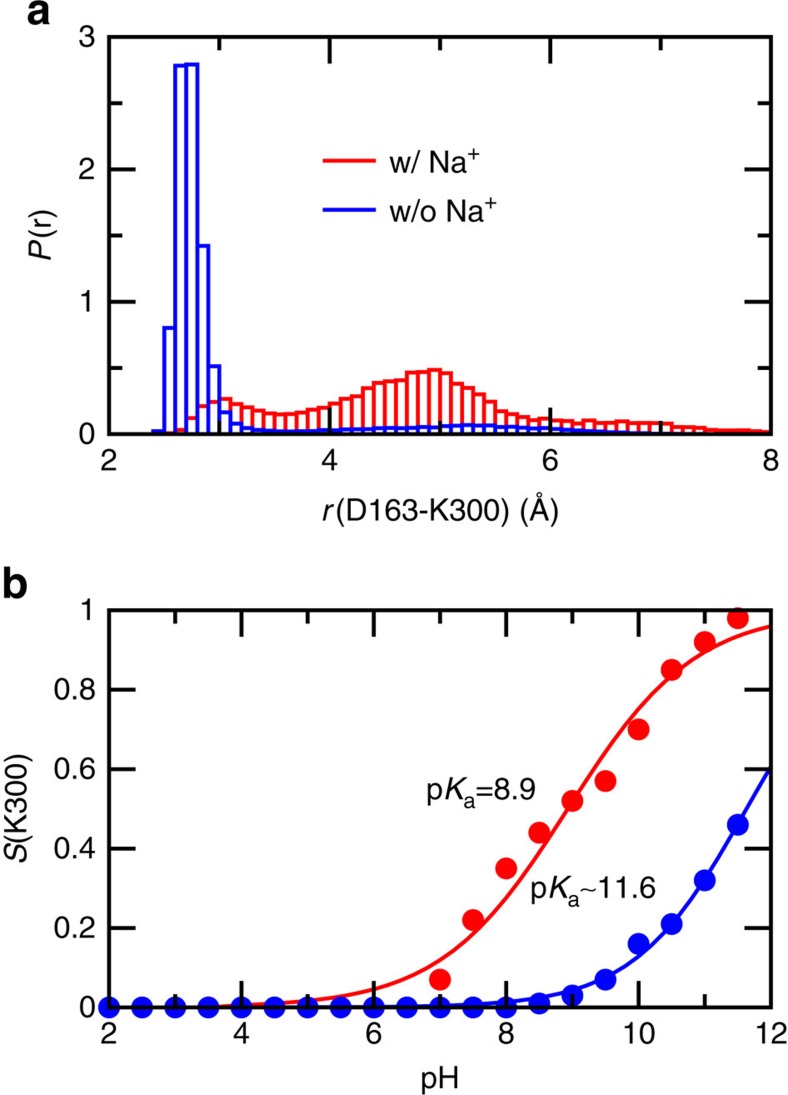
Sodium binding disrupts the Lys300–Asp163 salt bridge and lowers the p*K*_a_ of Lys300. (**a**) Probability distribution of the distance between the amine nitrogen of Lys300 and nearest carboxylate oxygen of Asp163 in the presence (red) and absence (blue) of sodium binding. Data from all pH conditions were used. (**b**) Fraction of deprotonated Lys300 at different pH in the presence (red) and absence (blue) of sodium binding. Solid curves are the best fits to the generalized Henderson–Hasselbalch equation. The estimated p*K*_a_'s are indicated. Sodium ion is considered bound if the distance to the nearest carboxylate oxygen of Asp163 is below 3 Å.

The identity of the second proton carrier has been controversial. Computational studies based on the earlier crystal structure pointed to Asp163 (refs 12, 36), which is also supported by the recent semi-macroscopic simulations based on the new crystal structure^24^. However, the recent MD simulation based on the new crystal structure suggested Lys300 (ref. 20), consistent with the experimental evidence that the p*K*_a_ of Lys300 is critical for the antiporter activity^11^. Our simulations initiated from both the recent and earlier crystal structures gave a macroscopic p*K*_a_ of around 10 for Lys300, which is only slightly below the model p*K*_a_ of 10.4 (model lysine in solution). The small p*K*_a_ shift is a result of the balance between the desolvation effect which decreases the p*K*_a_ and the electrostatic attraction with Asp163 which increases the p*K*_a_. Importantly, the latter is significantly influenced by ion binding. The simulation initiated from the recent crystal structure which contains the Lys300–Asp163 salt bridge showed that, in the presence of sodium binding to Asp163, the distance between Asp163 and Lys300 is significantly increased relative to configurations without sodium binding (Fig. 3a; Supplementary Fig. 6). Thus, sodium binding leads to the disruption of the Lys300–Asp163 salt bridge. Consequently, deprotonation of Lys300 is shifted to a lower pH range, and the (microscopic) p*K*_a_ in the presence of ion binding is reduced to 8.9, nearly three units from the p*K*_a_ of 11.6, calculated using configurations in the absence of sodium binding to Asp163 (Fig. 3b). Interestingly, experiment showed that the peak current increases at pH above 7 and reaches a maximum around pH 8.7 (refs 4, 5). Thus, our data suggest that within the active pH range Lys300 releases a proton, lending support to the most recent hypothesis that Lys300 is the second proton carrier^20^. We note that, while the calculated p*K*_a_ in the presence of sodium binding reports on the change in conformational environment, it does not account for the explicit ion interactions in the electrostatic calculations for propagating titration coordinates^33^,^42^, for example, between sodium and Lys300, which would destabilize the charged state of Lys300, thereby lowering its p*K*_a_. However, since the average distance between sodium and Lys300 is about 8 Å when sodium is bound to Asp163 (Supplementary Fig. 7), the p*K*_a_ decrease due to the latter effect is expected to be small.

Our calculated p*K*_a_ (11.6) in the absence of sodium binding shows an upshift from the model value, which is in qualitative agreement with the p*K*_a_ (12.81) obtained by the semi-macroscopic calculations^24^ (see Supplementary Information for detailed comparison), and suggests that Lys300 is charged in the inactive state of NhaA. To further validate the protonation state of Lys300, we examined its interactions with the partial negative dipoles of the C termini of TMs XIc and IVp. When Lys300 is charged, the interactions are intact; however, when Lys300 is neutralized, the interactions are disrupted (Supplementary Fig. 8). Thus, Lys300 is protonated in the inactive structure of NhaA. We note that, due to the limited timescale of MD simulations, the calculation of sodium flux was not attempted in this work. However, the study by Warshel and coworker based on MC simulations^24^ was able to provide the pH profile of sodium flux as well as the free energy profile of sodium binding, consistent with experiment^4^.

### Deprotonation of Asp164 triggers gate to open

**Figure 4:**
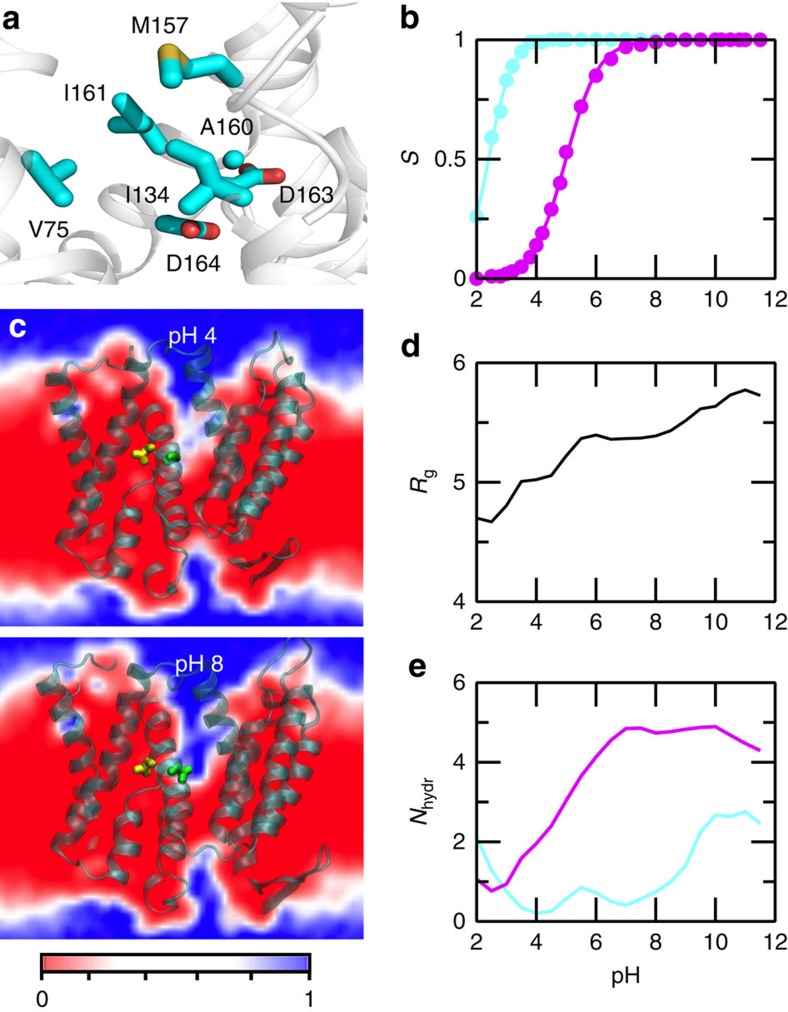
Deprotonation of Asp164 enables gate opening and water entrance. (**a**) A zoomed-in view of the cytoplasmic gate and core residues Asp163 and Asp164. (**b**) Fraction of deprotonated Asp163 (cyan) and Asp164 (magenta) as a function of pH. Solid curves represent fitting to the generalized Henderson–Hasselbalch equation. (**c**) Water density map at the cross section of Asp164 below (pH 4, upper panel) and above (pH 8, lower panel) the NhaA activation pH. Asp163 and Asp164 are shown as yellow and green spheres, respectively. The colour scale is based on the units of bulk density of TIP3P water at 298 K (ref. 68). The density map was generated using VMD^69^. (**d**) Radius of gyration of the cytoplasmic gate (V75, I134, M157, A160 and I161) as a function of pH. Calculation was based on the sidechain heavy atoms. (**e**) Hydration number of Asp163 (cyan) and Asp164 (magenta) as a function of pH. Hydration number refers to the number of water in the first solvation shell of Asp163/Asp164, defined using a cutoff distance of 3.5 Å between the water oxygen and the nearest carboxylate oxygen of Asp. In **d**,**e**, each data point (for clarity not explicitly shown) is an average over all frames at a given pH.

Having established that Asp164 is one of the proton carriers, and considering that it is the first core residue located immediately below the cytoplasmic funnel, we tested whether its deprotonation induces a conformational change and entrance of water. Opening of hydrophobic cavity and water penetration due to ionization of internal groups has been previously observed in both experimental and computational studies of Staphylococcal nuclease^39^,^43^,^44^. We zoom in on the end of the cytoplasmic funnel of NhaA, which narrows to a passage lined by five hydrophobic sidechains, Val75 (TM II), Ile134 (TM IV), Met157, Ala160 and Ile161 (TM V), before reaching the active site (Fig. 4a). These hydrophobic sidechains will be referred to as the cytoplasmic gate, since they control the access of water and ion from the cytoplasm. As Asp164 is located immediately below the gate with Asp163 next to it (Fig. 4a), we examined the titration behaviour of both residues (Fig. 4b; Supplementary Fig. 9) and the possible correlation with the passage opening and hydration of the core region.

To characterize the passage opening and hydration of the core, we calculated the radius of gyration based on the gate residues and the hydration number of Asp163 and Asp164 (number of water in the first solvation shell) as well as water density map (Fig. 4c–e; Supplementary Figs 10 and 11). At pH 2, Asp163 is partially and Asp164 is fully protonated; the radius of gyration is about 4.6 Å, similar to the crystal structure values, 4.2 Å of the earlier and 4.5 Å of the recent structure; there is one water near Asp163/Asp164. However, no ion is present in the core region (see later discussion). As pH is increased to 4, Asp163 becomes completely deprotonated (charged) and Asp164 remains protonated (neutral); the radius of gyration increases to about 5 Å. Interestingly, the hydration number of Asp164 increases to 2, whereas that of Asp163 remains below 1, and ions remain absent in the core region (later discussion). These data show that, charging of Asp163 leads to slight opening of the gate; however, the passage is not wide enough to accommodate a possibly hydrated sodium ion. Thus, at low pH the cytoplasmic funnel is closed, consistent with experiments showing NhaA is inactive either under a forward proton gradient with an inside pH below 6.5 (ref. 3) or under a sodium gradient with symmetric pH below 6.5 (ref. 4).

In the pH range 4–7, Asp163 remained deprotonated, while Asp164 titrates to become fully charged; there is a steep increase in both the radius of gyration and the hydration number of Asp164. At pH 7, the radius of gyration is about 5.5 Å, and the hydration number of Asp164 reaches 5, indicating that the gate is open and water enters, similar to the rapid wetting transition observed in hydrophobic nanopores of comparable size^45^,^46^. The pH-induced gate opening and water entrance into the core region is also visible from the water density map (Fig. 4c). In contrast, the hydration number of Asp163 remains very low. This is because Asp164 is immediately below the hydrophobic gate readily accessible to the cavity, whereas Asp163 is buried (Fig. 4c) and forms a salt bridge interaction with Lys300. Finally, as pH further increases above 7, there is little change in the radius of gyration or the hydration number of Asp164, which is readily understood since the deprotonation of Asp164 is complete at pH 7. In contrast, the hydration number of Asp163 sharply increases at pH above 8, which can be attributed to the disruption of the salt bridge with Lys300 following its deprotonation (Supplementary Fig. 12). Together, this set of data suggests that, while the deprotonation of Asp163 initiates local relaxation of the gate region, it is the deprotonation of Asp164 that triggers the gate to open so that water can enter the core region.

### Sodium binding to Asp163/Asp164/Thr132

**Figure 5:**
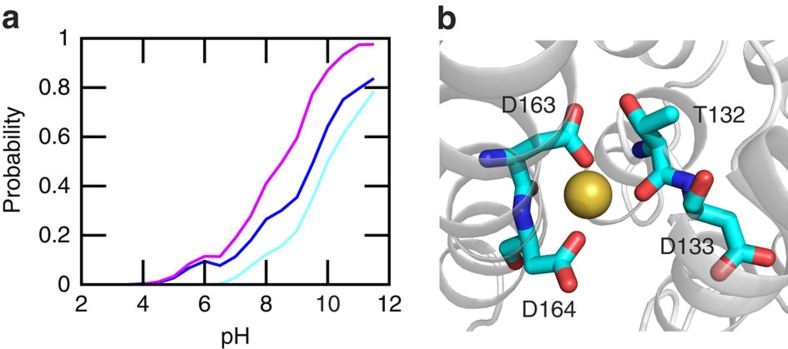
pH-dependent sodium binding to the core residues. (**a**) Probability of sodium binding to Thr132 (blue), Asp164 (magenta) and THr132/Asp163/Ap164 (cyan) as a function of pH. (**b**) A snapshot showing a sodium ion bound to Thr132/Asp163/Asp164. Sodium is considered bound if the distance to the nearest carboxylate oxygen of Asp163/Asp164 or the backbone carbonyl oxygen of T132 is below 3 Å. The probability was calculated by counting the number of frames. We note that the residence time of sodium is very long. Once it is bound, it does not leave in the CpHMD simulations. In the conventional simulations, the residence time is greater than 400 ns (ref. 20).

Along with the pH-induced gate dynamics and water penetration, our simulations reveal that a single sodium ion enters the cytoplasmic funnel and binds to the core residues in a pH-dependent manner. To identify the sodium binding sites, we calculated the probability of sodium binding based on a distance cutoff (Fig. 5a; Supplementary Fig. 13). Below pH 4, the probability of sodium binding to the core region is zero, in accord with the closed cytoplasmic gate and very limited water accessibility. Between pH 4 and 7, sodium occasionally coordinates to Asp164 and Thr132 through the carboxylate and backbone carbonyl groups, respectively, consistent with the opening of the cytoplasmic gate and water entrance. However, above pH 7, unlike the radius of gyration of the cytoplasmic gate or the hydration number of Asp164, which plateaus once the deprotonation of Asp164 is completed, the ion binding probability of Asp164 (and also Thr132) sharply increases. Moreover, Asp163 starts to bind sodium, resulting in the coordination to all three residues, Asp163, Asp164 and Thr132. These data suggest that the participation of Asp163 in ion binding stabilizes the ion residence in the core, facilitating the activation of NhaA above pH 7, consistent with experiments^3^,^4^. We note that the CpHMD data is consistent with the conventional fixed-protonation-state simulations showing that when Asp164 is deprotonated, sodium entered and bound to Asp163 and Asp164 (ref. 20). It is also in accord with the semi-macroscopic calculations which suggested that sodium binding is most favourable when both Asp163 and Asp164 are charged^24^.

### Deprotonation of Lys300 induces bending of TM V

**Figure 6:**
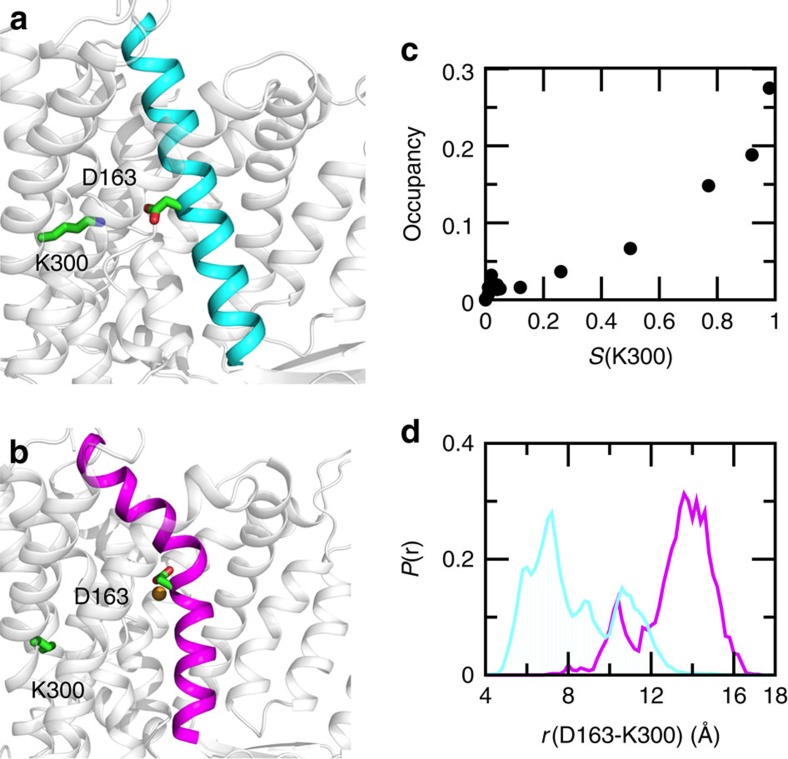
Deprotonation of Lys300 is correlated with bending of TM V. (**a**) Snapshot of NhaA with a straight TM V. (**b**) Snapshot of NhaA with a bent TM V. (**c**) Occupancy of the TM V-bent state versus fraction of the deprotonated Lys300. A configuration is considered as in the TM V-bent state if the TM V bending angle is greater than 28° (cutoff based on data shown in Supplementary Fig. 15). Data used all pH conditions (2.5–11.5). (**d**) Probability distribution of the minimum distance between the carboxylate oxygen of Asp163 and the amine nitrogen of Lys300 when TM V is bent (magenta) and straight (cyan). The data are from pH conditions 9–11.5 where TM V bending was observed. In all panels, the simulation run 2 starting from the previous crystal structure (PDB ID: 1ZCD) was used.

An important aspect of the sodium-proton exchange process involves a conformational change, presumably following the release of two protons in exchange for a sodium. Such a conformational change is expected to occur on a much longer timescale as compared to our simulations. However, surprisingly, in one set of the CpHMD simulations (run 2 initiated from the earlier crystal structure), we observed that TM V, which resides between the core and dimerization domains, exhibited a bend around Asp163 when Lys300 is deprotonated (Fig. 6a,b). In addition, principal component analysis revealed that the major mode of the inward-facing NhaA involves a motion of the core relative to the dimerization domain (Supplementary Fig. 14). To quantify the degree of TM V bending, a bending angle is defined as the deviation from linearity of the angle formed by the C*α* atoms of Leu152 (one end of TM V), Asp163 (middle of TM V) and Phe174 (the other end of TM V). Below pH 7 (activation pH), the peak of the bending angle distribution, that is, the most probable angle, is between 0° and 10°, indicating that TM V is nearly straight (Supplementary Fig. 15). At pH 7–10 (Asp164 is deprotonated and Lys300 is protonated), the distribution broadens to the range 5–22°, indicating that TM V becomes more flexible. Remarkably, as pH is further increased such that Lys300 becomes deprotonated, the distribution becomes bimodal with a second peak centring at about 34°, indicating the emergence of a second conformational state with a bent TM V.

Plotting the occupancy of the TM V-bent state versus the fraction of Lys300 deprotonation reveals a nearly linear relationship (Fig. 6c), that is, the deprotonation of Lys300 is correlated with TM V bending. Further trajectory analysis revealed that in the TM V-bent state, Asp163 is bound to a sodium ion, while Lys300 is deprotonated and rotated away with a significantly increased distance between the two (Fig. 6b,d). These data suggest that the release of a second proton from Lys300 and the accompanying sodium binding of Asp163 lead to the bending of TM V. We note that, although TM V bending was not observed in the other two sets of CpHMD simulations, likely due to the limited sampling time, it was also seen in the conventional simulation initiated from the recent crystal structure and with Asp163/Asp164/Lys300 fixed in the deprotonated state (Supplementary Fig. 15). To further understand the origin of TM V bending, we examined the dynamics of TM V and its interactions with the environment. We found that the bending is correlated with the breakage of a hydrogen bond between the carboxylate of Asp163 and the backbone amide of Thr132 located on the adjacent helix. When TM V is straight, the hydrogen bond is intact; however, when TM V bends, the hydrogen bond is disrupted (Supplementary Fig. 16). Because the bend is located at Asp163, the Thr132–Asp163 hydrogen bond appears to hold the helix in place.

### Agreement between CpHMD and conventional simulations

A major limitation of the current CpHMD implementation is the relatively short timescale (on the order of 10 ns per replica), which may result in the incomplete sampling of conformational states of protein and solvent despite the use of the pH replica-exchange enhanced sampling protocol. To assess convergence, we compare key quantities with the conventional simulations for different combinations of protonation states (1–3 μs each). Agreement is observed for the hydration levels for Asp163, and Asp164, the probability of sodium binding to Asp163, Asp164 and Thr132, and the behaviour of Asp163–Lys300 interaction (Supplementary Note 2; Supplementary Figs 17–20). As to the latter, the new NhaA crystal structure solved at pH 3.5 shows a salt bridge^20^, which remains stable in the conventional simulations S1 and S2 with charged Asp163 and Lys300. Consistent with these data, the CpHMD simulations now clearly explain this observation as a consequence of the low p*K*_a_ of Asp163. The consistency between low pH crystal structure and both sets of simulations also corroborates the hypothesis that Asp163 is not one of the proton carriers because it remains charged at all physiologically accessible pH conditions.

## Discussion

A key question regarding the mechanism of NhaA is related to the identities of specific residues responsible for proton uptake and release in the transport cycle. For a number of years, Asp163 and Asp164 have been considered as the two proton carriers^4^,^6^,^17^,^36^. However, recent molecular dynamics simulations based on the new crystal structure of NhaA suggested Lys300 but not Asp163 is the second proton carrier^20^, consistent with biochemical data^11^. Our results support the latter model. The CpHMD simulations showed that, when Lys300 forms a salt bridge with Asp163, its p*K*_a_ is high (11.6) due to stabilization of the charged state by the attractive Coulomb interaction; however, when a sodium ion binds to Asp163, Lys300 rotates away leading to disruption of the salt bridge and lowering of the Lys300 p*K*_a_ by almost 3 units to 8.9. We note that, the latter p*K*_a_ is likely somewhat overestimated due to a known limitation of the hybrid-solvent CpHMD method which neglects the explicit interactions with ions^33^,^42^ which would destabilize the charged state of Lys300, lowering its p*K*_a_ further. Thus, it is very likely that as pH reaches above 7–8 the release of the second proton from Lys300 could occur simultaneously with sodium binding to Asp163.

A second hypothesis our study aimed to test is related to a single residue at the entrance of the cytoplasmic funnel (pH sensor), which is thought to sense the pH signal and regulate the activation of NhaA allosterically^15^. An earlier study based on the continuum electrostatics calculations suggested that Glu78 has a p*K*_a_ near the physiological range and can therefore trigger a conformational change required for activation^12^. Our simulations do not support this conjecture, as the calculated p*K*_a_'s of all acidic residues including Glu78 in the pH sensor are far below the physiological pH. Instead, three histidines in the pH sensor have p*K*_a_'s in the physiological range, and the switch of their protonation states together changes the net charge of the pH sensor from positive to negative at around pH 7, the same pH range in which sizable transporter activity could be experimentally measured^3^,^4^. Thus, we propose that the pH sensor residues collectively respond to the pH signal and attract sodium ions when pH rises above the activation value. This hypothesis is consistent with the observations that mutations of all the pH sensor residues strongly influence the pH-dependent activity of NhaA^14^,^15^,^16^.

Until now two models have been put forth to explain the pH regulation of NhaA. In the allosteric model proposed by Padan and coworkers^1^,^15^, a pH sensor residue (located at the cytoplamic funnel entrance but far from the core region) senses the pH signal and induces a conformational change that activates NhaA. In the competitive binding model^4^,^19^, a sodium ion and two protons compete for binding to the active site. Substrate binding lowers the energy barrier between two conformational states, resulting in the transition from one state to the other. Our data showed that, above the activation pH the deprotonation of Asp164 triggers the opening of the cytoplasmic gate formed by several hydrophobic residues lining the bottom of the cytoplasmic funnel and entrance of water as well as ion to the core region in a pH-dependent manner. Thus, access to the binding site is controlled via a pH-dependent hydrophobic gating mechanism^45^,^46^,^47^. Further, our simulations suggested that, sodium binding to Asp163 can trigger the release of a proton from Lys300, which in turn can induce bending of TM V located between the core and dimerization domains. Although such movement is not seen in the outward-facing state of NapA^21^ and MjNhaP1 from archaea^48^, we hypothesize that, since our simulations only capture the initial stage of the transport cycle, TM V bending may represent a kinetic intermediate accompanying the large conformational transition to the outward-facing state. Interestingly, it is consistent with the electrophysiology data which showed that mutation A167P on helix V markedly slows the rate of conformational transition but does not affect the optimum pH of NhaA^5^. To directly verify TM V bending, we suggest to test the mutation Thr132 to Val132, which eliminates the hydrogen bond with Asp163, would possibly facilitate TM V bending and accelerate the conformational switch. Thus, our data lend support to the competitive binding model for NhaA, modulated with the two pH-dependent effects of electrostatic funneling due to the overall charge of the pH sensor and the hydrophobic gate controlling access to the binding site.

Taken together, our work suggests the following mechanism for the antiport activity of NhaA. As pH is increased to the activation pH, the net charge of the pH sensor residues decreases to a negative value, attracting a sodium ion into the funnel. At the same time, Asp164 releases the first proton which induces opening of the cytoplasmic hydrophobic gate. The latter allows the entrance of water and a sodium ion, which is first captured by Asp164, the residue immediately below the cytoplasmic gate, and subsequently shared with Asp163 and Thr132. Sodium binding to Asp163 disrupts the salt bridge with Lys300, destabilizing its charged state and leading to the release of the second proton. The latter triggers a conformational change, possibly involving bending of TM V, which may precede a large conformational transition^22^ to the outward-facing state of NhaA. Because of the short simulation timescale, the above mechanism describes only the early events of the transport cycle. Nonetheless, our work reconciles the current models and provides atomic details of the pH-dependent activation and sodium-proton antiport of NhaA. Finally, we note that the CpHMD methodology is general and can be applied to illuminate other proton-coupled conformational processes in biology that are difficult to delineate by current experimental techniques.

## Methods

### System preparation

Two crystal structures of NhaA (PDB IDs: 1ZCD^6^ and 4AU5 (ref. 20)) were employed for this study. CpHMD run 1 was initiated from the monomer B of the new crystal structure (PDB ID: 4AU5, sequence 10–383), while run 2 and 3 were initiated from the subunit A of the previous crystal structure which is a monomer (PDB ID: 1ZCD, sequence 10–383 was used to be consistent with the monomer B of the new crystal structure). Hydrogen atoms were added to the protein using the HBUILD facility in CHARMM. The protein were then inserted into a preassembled lipid bilayer with 135 1-palmitoyl-2-oleoyl-sn-glycero-3-phosphocholine (POPC) lipids using Membrane Builder in CHARMM-GUI (http://www.charmm-gui.org)^49^. The resulting number of lipids is 63 for the cytoplasmic- and 72 for the periplasm-facing leaflet. A water layer of 15 Å thickness was added to both sides of the lipid bilayer. Twenty-one sodium and 30 chloride ions for 4AU5 or 20 sodium and 29 chloride ions for 1ZCD were added to neutralize the simulation box at neutral pH (assuming model p*K*_a_'s for Asp/Glu/His/Lys) and to reach the ionic strength of 150 mM. To prepare for the continuous constant pH molecular dynamics (CpHMD) simulations dummy hydrogen atoms were added to the carboxylate groups of acidic residues following the documentation of the PHMD module^31^,^32^ in CHARMM^50^. The final systems contain about 50,000 atoms.

### CpHMD simulations with pH replica exchange

The work employed the membrane-embedded hybrid-solvent CpHMD method^32^,^33^,^51^ with pH replica-exchange sampling protocol^33^. Here conformational sampling of transmembrane proteins is performed in explicit lipid bilayer, as in the conventional all-atom MD. The generalized Born GBSW membrane model^40^,^52^ is used to efficiently calculate the solvation free energies and forces needed to propagate titration coordinates. The pH replica-exchange protocol is employed to accelerate the convergence of p*K*_a_'s and pH-dependent conformational sampling^33^. The hybrid-solvent CpHMD has been demonstrated to offer rapid and accurate prediction of p*K*_a_'s and conformational dynamics involving sites buried in the protein interior as well as surfactant and lipid environments^39^,^42^,^53^.

CpHMD simulations were performed using CHARMM programme package (version c37a2)^50^, where the CpHMD method (PHMD module)^32^,^33^,^51^ and pH replica-exchange protocol (REPDSTR module)^33^ were implemented. The CHARMM22/CMAP all-atom force field^54^,^55^ was used to represent the protein, while the CHARMM36 lipid force field^56^ was used to represent the lipids. Water molecules were represented by the CHARMM modified TIP3P water model^50^. For propagation of titration coordinates, the membrane GBSW implicit-solvent model was used^40^,^52^. The GBSW input radii for the protein were taken from Chen *et al*.^57^. The implicit membrane thickness was set to 30 Å, which was calculated as the average distance between the C2 atoms of the lipids in the cytoplasmic- and periplasmic-facing leaflets. A switching region of 5 Å thickness was used for the transition between the low dielectric slab and bulk solvent. To exclude the implicit membrane from the interior of the protein, an exclusion cylinder with a radius of 15 Å was placed at the center of mass of the protein. The radius was chosen such that the cylinder encompasses the entire protein and minimal number of lipids. Other settings in CpHMD and GBSW follow the default values in the programme (see documentation of CHARMM c37a2 (ref. 50)).

The system was first equilibrated at pH 4 (near the crystallization pH condition) for 2.4–5.4 ns using the default multi-step protocol in CHARMM-GUI^49^,^58^. Following equilibration, the production simulation was performed using hybrid-solvent CpHMD with the pH replica-exchange protocol^33^, whereby each replica was simulated under constant NPTpH conditions at 310 K and 1 atm and specified pH. Temperature was maintained using a modified Hoover thermostat method^59^, while pressure was maintained using the Langevin piston coupling method^60^. The van der Waals interactions were smoothly switched to zero between 10 and 12 Å. The particle mesh Ewald method^61^ was used to calculate long-range electrostatics, with a real-space cutoff of 12 Å and a sixth-order interpolation with 0.9 Å grid spacing. The SHAKE algorithm was used to constrain bonds involving hydrogen to enable a 2-fs timestep. A GBSW electrostatic solvation free energy calculation^40^,^52^ was invoked every 5 MD steps to update the titration coordinates. The default settings were used, consistent with our previous work^33^. For simulation run 1, the pH range was 1.5–11.5 with a total of 28 replicas. For simulation run 2 and 3, the pH range was 2.5–11.5 with a total of 28 replicas. The specific pH conditions for run 1 were 1.5, 2.0, 2.5, 2.75, 3.0, 3.25, 3.5, 3.75, 4.0, 4.25, 4.5, 4.75, 5.0, 5.5, 6, 6.5, 7, 7.5, 8.0, 8.5, 9.0, 9.5, 10.0, 10.25, 10.5, 10.75, 11.0 and 11.5. The specific pH conditions for run 2 and 3 were 2.5, 2.75, 3.0, 3.25, 3.5, 3.75, 4.0, 4.25, 4.5, 4.75, 5.0, 5.5, 6, 6.5, 6.75, 7, 7.25, 7.5, 8.0, 8.5, 9.0, 9.5, 10.0, 10.25, 10.5, 10.75, 11.0 and 11.5. An exchange between adjacent pH conditions was attempted every 500 MD steps (or 1 ps) with an acceptance ratio of about 30% for all replicas. The production simulation lasted 10–14 ns per replica, resulting in an aggregate sampling time of 280–381 ns for each set of CpHMD simulations. Data from the last 4 ns per replica for simulation runs 1 and 3 or the last 5.6 ns per replica for simulation run 2 was used for analysis. We note that within the production run time, the p*K*_a_ of relevant residues were converged. Although longer simulation time may be desirable, we were constrained by the current speed of the CpHMD implementation (in CHARMM) and the available hardware resources.

### Conventional fixed-protonation-state simulations

Simulations with fixed protonation states were taken from previous work by Lee *et al*.^20^. In brief, all-atom explicit solvent MD simulations were run with GROMACS^62^,^63^ with the OPLS-AA force field for protein and ions, and the TIP4P model for water^64^. The force field for POPC lipids was taken from Ulmschneider and Ulmschneider^65^. The orthorhombic simulation cell contained a NhaA dimer and about 112,000 atoms. Simulations were performed in the NPT ensemble with PME electrostatics and a 2-fs timestep while constraining all bonds including hydrogen atoms. The protonation states of key residues were manually fixed for each simulation as detailed in Supplementary Table 1; other residues were set to default values (see ref. 20 for further details). Simulations S1, S2, S4 were repeated three times and analysed in aggregate (totalling between 1.4 and 3 μs for each charge configuration); for S3 only a single 1-μs simulation was analysed (Supplementary Table 1). Trajectories were analysed at 1 ns intervals.

To check for a significant dependence on lipids, we performed a second, smaller set of simulations using a 4:1 POPE:POPG membrane which approximates the composition of a native *E. coli* inner membrane (see Table 1 for further simulation details). The simulations utilized the CHARMM36 protein^66^ and CHARMM36 lipid force fields^56^, the default CHARMM sodium ion parameters^67^ and CHARMM TIP3P water model^50^. The simulations were performed with the same protocol that was previously employed for the simulations of NapA^22^.

### Analysis

The p*K*_a_ values were calculated by fitting the unprotonated fractions of specified residue at simulated pH conditions to the generalized Henderson–Hasselbalch equation, 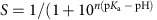), where *S* is the unprotonated fraction and *n* is the Hill coefficient.

A Cα-atom-based principal component analysis was performed using the VIBRAN module in CHARMM^50^. The analysis was carried out for the trajectory at pH 11.5 from simulation run 2 in which bending of TM V was observed (see main text). The average coordinates were used as the reference. The first principal component has a larger eigenvalue than others, which accounts for 32% of the total variance.

### Data availability

Molecular dynamics parameters, inputs and initial coordinates are available for download at http://drum.lib.umd.edu/handle/1903/18477. All other data are available from the corresponding author upon reasonable request.

## Additional information

**How to cite this article:** Huang, Y. *et al*. Mechanism of pH-dependent activation of the sodium-proton antiporter NhaA. *Nat. Commun.* **7,** 12940 doi: 10.1038/ncomms12940 (2016).

## Supplementary Material

Supplementary Information Supplementary Figures 1-20, Supplementary Tables 1-2, Supplementary Notes 1-2 and Supplementary References

Peer review file

## Figures and Tables

**Table 1:** Calculated p*K*_a_'s of titratable residues in the pH sensor and active site of NhaA.

Residue	p*K*_a_*	Residue	p*K*_a_*
*pH sensor*			
Asp11	2.6 (<2.5)	His243	6.8 (6.9/7.0)
Glu78	3.1 (3.7/3.6)	His253	6.3 (6.5/6.3)
Glu82	3.3 (3.2/2.6)	His256	6.9 (7.2/7.0)
Glu252	2.6 (3.1/2.7)	Lys249	11.2 (11.1/11.0)
*Active site*			
Asp133	4.5 (4.7/4.4)	Asp163	2.4 (3.5/3.9)
Lys300	10.1† (10.0/10.0)	Asp164	5.0 (6.6/5.8)

^*^The p*K*_a_'s calculated from run 2 and 3 starting from the previous crystal structure (PDB ID: 1ZCD) are listed in parenthesis.

^†^The macroscopic p*K*_a_ based on all configurations. When separating configurations in the presence and absence of sodium binding to Asp163, the calculated p*K*_a_'s are 8.9 and 11.6, respectively (Fig. 3a,b). The latter is approximate as the deprotonation is incomplete at the highest simulation pH 11.5. The p*K*_a_'s of all titratable sites are given in Supplementary Table 2.

## References

[b1] Padan E. Functional and structural dynamics of NhaA, a prototype for Na^+^ and H^+^ antiporters, which are responsible for Na^+^ and H^+^ homeostasis in cells. Biochim. Biophys. Acta 1837, 1047–1062 (2014). 24361841 10.1016/j.bbabio.2013.12.007

[b2] Padan E., Bibi E., Ito M. & Krulwich T. A. Alkaline pH homeostasis in bacteria: new insights. Biochim. Biophys. Acta 1717, 67–88 (2005). 16277975 10.1016/j.bbamem.2005.09.010 PMC3072713

[b3] Taglicht D., Padan E. & Schuldiner S. Overproduction and purification of a functional Na^+^/H^+^ antiporter coded by nhaA (ant) from *Escherichia coli*. J. Biol. Chem. 266, 11289–11294 (1991). 1645730

[b4] Mager T., Rimon A., Padan E. & Fendler K. Transport mechanism and pH regulation of the Na^+^/H^+^ antiporter NhaA from *Escherichia coli*. J. Biol. Chem. 286, 23570–23581 (2011). 21566125 10.1074/jbc.M111.230235 PMC3123120

[b5] Mager T. . Differential effects of mutations on the transport properties of the Na^+^/H^+^ antiporter NhaA from *Escherichia coli*. J. Biol. Chem. 288, 24666–24675 (2013). 23836890 10.1074/jbc.M113.484071 PMC3750164

[b6] Hunte C. . Structure of a Na^+^/H^+^ antiporter and insights into mechanism of action and regulation by pH. Nature 435, 1197–1202 (2005). 15988517 10.1038/nature03692

[b7] Inoue H., Noumi T., Tsuchiya T. & Kanazawa H. Essential aspartic acid residues, Asp-133, Asp-163 and Asp-164, in the transmembrane helices of a Na^+^/H^+^ antiporter (NhaA) from *Escherichia coli*. FEBS Lett. 363, 264–268 (1995). 7737413 10.1016/0014-5793(95)00331-3

[b8] Galili L., Rothman A., Kozachkov L., Rimon A. & Padan E. Transmembrane domain IV is involved in ion transport activity and pH regulation of the NhaA-Na^+^/H^+^ antiporter of *Escherichia coli*. Biochemistry 41, 609–617 (2002). 11781101 10.1021/bi011655v

[b9] Galili L., Herz K., Dym O. & Padan E. Unraveling functional and structural interactions between transmembrane domains IV and XI of NhaA Na^+^/H^+^ antiporter of *Escherichia coli*. J. Biol. Chem. 279, 23104–23113 (2004). 15039449 10.1074/jbc.M400288200

[b10] Kozachkov L., Herz K. & Padan E. Functional and structural interactions of the transmembrane domain X of NhaA, Na^+^/H^+^ antiporter of *Escherichia coli*, at physiological pH. Biochemistry 46, 2419–2430 (2007). 17284054 10.1021/bi602393s

[b11] Maes M., Rimon A., Kozachkov-Magrisso L., Friedler A. & Padan E. Revealing the ligand binding site of NhaA Na^+^/H^+^ antiporter and its pH dependence. J. Biol. Chem. 287, 38150–38157 (2012). 22915592 10.1074/jbc.M112.391128 PMC3488084

[b12] Olkhova E., Hunte C., Screpanti E., Pandan E. & Michel H. Multiconformation continuum electrostatics analysis of the NhaA Na^+^/H^+^ antiporter of Escherichia coli with functional implications. Proc. Natl Acad. Sci. USA 103, 2629–2634 (2006). 16477015 10.1073/pnas.0510914103 PMC1413810

[b13] Olkhova E., Kozachkov L., Padan E. & Michel H. Combined computational and biochemical study reveals the importance of electrostatic interactions between the ‘pH sensor' and the cation binding site of the sodium/proton antiporter NhaA of *Escherichia coli*. Proteins 76, 548–559 (2009). 19274728 10.1002/prot.22368

[b14] Tzubery T., Rimon A. & Padan E. Structure-based functional study reveals multiple roles of transmembrane segment IX and loop VIII-IX in NhaA Na^+^/H^+^ antiporter of *Escherichia coli* at physiological pH. J. Biol. Chem. 283, 15975–15987 (2008). 18387952 10.1074/jbc.M800482200 PMC3259659

[b15] Padan E. The enlightening encounter between structure and function in the NhaA Na^+^-H^+^ antiporter. Trends Biochem. Sci. 33, 435–443 (2008). 18707888 10.1016/j.tibs.2008.06.007

[b16] Herz K., Rimon A., Olkhova E., Kozachkov L. & Padan E. Transmembrane segment II of NhaA Na^+^/H^+^ antiporter lines the cation passage, and Asp65 is critical for pH activation of the antiporter. J. Biol. Chem. 285, 2211–2220 (2010). 19923224 10.1074/jbc.M109.047134 PMC2804377

[b17] Kozachkov L. & Padan E. Site-directed tryptophan fluorescence reveals two essential conformational changes in the Na^+^/H^+^ antiporter NhaA. Proc. Natl Acad. Sci. USA 108, 15769–15777 (2011). 21873214 10.1073/pnas.1109256108 PMC3179058

[b18] Călinescu O., Paulino C., Kühlbrandt W. & Fendler K. Keeping it simple, transport mechanism and pH regulation in Na^+^/H^+^ exchangers. J. Biol. Chem. 289, 13168–13176 (2014). 24644283 10.1074/jbc.M113.542993 PMC4036328

[b19] Călinescu O. & Fendler K. A universal mechanism for transport and regulation of CPA sodium proton exchangers. Biol. Chem. 396, 1091–1096 (2015). 25719314 10.1515/hsz-2014-0278

[b20] Lee C. . Crystal structure of the sodium-proton antiporter NhaA dimer and new mechanistic insights. J. Gen. Physiol. 144, 529–544 (2014). 25422503 10.1085/jgp.201411219 PMC4242812

[b21] Lee C. . A two-domain elevator mechanism for sodium/proton antiport. Nature 501, 573–577 (2013). 23995679 10.1038/nature12484 PMC3914025

[b22] Coincon M. . Crystal structures reveal the molecular basis of ion translocation in sodium/proton antiporters. Nat. Struct. Mol. Biol. 23, 248–255 (2016). 26828964 10.1038/nsmb.3164

[b23] Søndergaard C. R., Mats H. M. Olsson M. R. & Jensen J. H. Improved treatment of ligands and coupling effects in empirical calculation and rationalization of pKa values. J. Chem. Theory Comput. 7, 2284–2295 (2011). 26606496 10.1021/ct200133y

[b24] Alhadeff R. & Warshel A. Simulating the function of sodium/proton antiporters. Proc. Natl Acad. Sci. USA 112, 12378–12383 (2015). 26392528 10.1073/pnas.1516881112 PMC4603454

[b25] Baptista A. M., Teixeira V. H. & Soares C. M. Constant-pH molecular dynamics using stochastic titration. J. Chem. Phys. 117, 4184–4200 (2002).

[b26] Mongan J., Case D. A. & McCammon J. A. Constant pH molecular dynamics in generalized Born implicit solvent. J. Comput. Chem. 25, 2038–2048 (2004). 15481090 10.1002/jcc.20139

[b27] Swails J. M. & Roitberg A. E. Enhancing conformation and protonation state sampling of hen egg white lysozyme using pH replica exchange molecular dynamics. J. Chem. Theory Comput. 8, 4393–4404 (2012). 26605601 10.1021/ct300512h

[b28] Lee J., Miller B. T., Damjanović A. & Brooks B. R. Constant pH molecular dynamics in explicit solvent with enveloping distribution sampling and Hamiltonian exchange. J. Chem. Theory Comput. 10, 2738–2750 (2014). 25061443 10.1021/ct500175m PMC4095908

[b29] Chen W., Morrow B. H., Shi C. & Shen J. K. Recent development and application of constant pH molecular dynamics. Mol. Simul. 40, 830–838 (2014). 25309035 10.1080/08927022.2014.907492 PMC4188395

[b30] Messer B. M. . Multiscale simulations of protein landscapes: using coarse-grained models as reference potentials to full explicit models. Proteins 78, 1212–1227 (2010). 20052756 10.1002/prot.22640 PMC2822134

[b31] Lee M. S., Salsbury F. R. Jr & Brooks C. L. III. Constant-pH molecular dynamics using continuous titration coordinates. Proteins 56, 738–752 (2004). 15281127 10.1002/prot.20128

[b32] Khandogin J. & Brooks C. L. III. Constant pH molecular dynamics with proton tautomerism. Biophys. J. 89, 141–157 (2005). 15863480 10.1529/biophysj.105.061341 PMC1366513

[b33] Wallace J. A. & Shen J. K. Continuous constant pH molecular dynamics in explicit solvent with pH-based replica exchange. J. Chem. Theory Comput. 7, 2617–2629 (2011). 26606635 10.1021/ct200146j PMC6425487

[b34] Dani J. A. Ion-channel entrances influence permeation netcharge, shape, and binding considerations. Biophys. J. 49, 607–618 (1986). 2421791 10.1016/S0006-3495(86)83688-8 PMC1329508

[b35] Gonzales E. B., Kawate T. & Gouaux E. Pore architecture and ion sites in acid-sensing ion channels and P2X receptors. Nature 460, 599–604 (2009). 19641589 10.1038/nature08218 PMC2845979

[b36] Arkin I. T. . Mechanism of Na^+^/H^+^ antiporting. Science 317, 799–803 (2007). 17690293 10.1126/science.1142824

[b37] Gunner M. R., Zhu X. & Klein M. C. MCCE analysis of the p*K*_*a*_s of introduced buried acids and bases in staphylococcal nuclease. Proteins 79, 3306–3319 (2011). 21910138 10.1002/prot.23124

[b38] Schutz C. N. & Warshel A. What are the dielectric constants of proteins and how to validate electrostatic models? Proteins 44, 400–417 (2001). 11484218 10.1002/prot.1106

[b39] Shi C., Wallace J. A. & Shen J. K. Thermodynamic coupling of protonation and conformational equilibria in proteins: theory and simulation. Biophys. J. 102, 1590–1597 (2012). 22500759 10.1016/j.bpj.2012.02.021 PMC3318114

[b40] Im W., Lee M. S. & Brooks C. L. III. Generalized Born model with a simple smoothing function. J. Comput. Chem. 24, 1691–1702 (2003). 12964188 10.1002/jcc.10321

[b41] Wallace J. A. . Toward accurate prediction of p*K*_*a*_ values for internal protein residues: the importance of conformational relaxation and desolvation energy. Proteins 79, 3364–3373 (2011). 21748801 10.1002/prot.23080

[b42] Morrow B. H., Eike D. M., Murch B. P., Koenig P. H. & Shen J. K. Predicting proton titration in cationic micelle and bilayer environments. J. Chem. Phys. 141, 084714 (2014). 25173037 10.1063/1.4893439 PMC4149686

[b43] Kato M. & Warshel A. Using a charging coordinate in studies of ionization induced partial unfolding. J. Phys. Chem. B 110, 11566–11570 (2006). 16771433 10.1021/jp061190o

[b44] Damjanović A., Brooks B. R. & Garca-Moreno E. B. Conformational relaxation and water penetration coupled to ionization of internal groups in proteins. J. Phys. Chem. A 115, 4042–4053 (2011). 21428436 10.1021/jp110373f PMC3373309

[b45] Beckstein O., Biggin P. C. & Sansom M. S. P. A hydrophobic gating mechanism for nanopores. J. Phys. Chem. B 105, 12902–12905 (2001).

[b46] Beckstein O. & Sansom M. S. P. The influence of geometry, surface character, and flexibility on the permeation of ions and water through biological pores. Phys. Biol. 1, 42–52 (2004). 16204821 10.1088/1478-3967/1/1/005

[b47] Aryal P., Sansom M. S. & Tucker S. J. Hydrophobic gating in ion channels. J. Mol. Biol. 427, 121–130 (2015). 25106689 10.1016/j.jmb.2014.07.030 PMC4817205

[b48] Paulino C., Wöhlert D., Kapotova E., Yildiz Ö. & Kühlbrandt W. Structure and transport mechanism of the sodium/proton antiporter MjNhaP1. eLife 3, e03583 (2014). 25426803 10.7554/eLife.03583 PMC4381896

[b49] Wu E. L. . CHARMM-GUI *Membrane Builder* toward realistic biological membrane simulations. J. Comput. Chem. 35, 1997–2004 (2014). 25130509 10.1002/jcc.23702 PMC4165794

[b50] Brooks B. R. . CHARMM: the biomolecular simulation program. J. Comput. Chem. 30, 1545–1614 (2009). 19444816 10.1002/jcc.21287 PMC2810661

[b51] Khandogin J. & Brooks C. L. III. Toward the accurate first-principles prediction of ionization equilibria in proteins. Biochemistry 45, 9363–9373 (2006). 16878971 10.1021/bi060706r

[b52] Im W., Feig M. & Brooks C. L. III. An implicit membrane generalized Born theory for the study of structure, stability, and interactions of membrane proteins. Biophys. J. 85, 2900–2918 (2003). 14581194 10.1016/S0006-3495(03)74712-2 PMC1303570

[b53] Ellis C. R. & Shen J. pH-dependent population shift regulates BACE1 activity and inhibition. J. Am. Chem. Soc. 137, 9543–9546 (2015). 26186663 10.1021/jacs.5b05891 PMC4697946

[b54] MacKerell A. D. Jr . All-atom empirical potential for molecular modeling and dynamics studies of proteins. J. Phys. Chem. B 102, 3586–3616 (1998). 24889800 10.1021/jp973084f

[b55] MacKerell A. D. Jr, Feig M. & Brooks C. L. III. Extending the treatment of backbone energetics in protein force fields: limitations of gas-phase quantum mechanics in reproducing protein conformational distributions in molecular dynamics simulations. J. Comput. Chem. 25, 1400–1415 (2004). 15185334 10.1002/jcc.20065

[b56] Klauda J. B. . Update of the CHARMM all-atom additive force field for lipids: validation on six lipid types. J. Phys. Chem. B 114, 7830–7843 (2010). 20496934 10.1021/jp101759q PMC2922408

[b57] Chen J., Im W. & Brooks C. L. III. Balancing solvation and intramolecular interactions: toward a consistent generalized Born force field. J. Am. Chem. Soc. 128, 3728–3736 (2006). 16536547 10.1021/ja057216r PMC2596729

[b58] Jo S., Kim T. & Im W. Automated builder and database of protein/membrane complexes for molecular dynamics simulations. PLoS ONE 2, e880 (2007). 17849009 10.1371/journal.pone.0000880 PMC1963319

[b59] Hoover W. G. Canonical dynamics: equilibration phase-space distributions. Phys. Rev. A 31, 1695–1697 (1985). 10.1103/physreva.31.1695 9895674

[b60] Feller S. E., Zhang Y., Pastor R. W. & Brooks B. R. Constant pressure molecular dynamics simulation: the Langevin piston method. J. Chem. Phys. 103, 4613–4621 (1995).

[b61] Darden T., York D. & Pedersen L. Particle mesh Ewald: an N log(N) method for Ewald sums in large systems. J. Chem. Phys. 98, 10089–10092 (1993).

[b62] Pronk S. . GROMACS 4.5: a high-throughput and highly parallel open source molecular simulation toolkit. Bioinformatics 29, 845–854 (2013). 23407358 10.1093/bioinformatics/btt055 PMC3605599

[b63] Páll S., Abraham M. J., Kutzner C., Hess B. & Lindahl E. Solving Software Challenges for Exascale, chap. Tackling Exascale Software Challenges in Molecular Dynamics Simulations with GROMACS 3–27Springer (2015).

[b64] Jorgensen W. L. & Tirado-Rives J. Potential energy functions for atomic-level simulations of water and organic and biomolecular systems. Proc. Natl Acad. Sci. USA 102, 6665–66670 (2005). 15870211 10.1073/pnas.0408037102 PMC1100738

[b65] Ulmschneider J. P. & Ulmschneider M. B. United atom lipid parameters for combination with the pptimized potentials for liquid simulations all-atom force field. J. Chem. Theory Comput. 5, 1803–1813 (2009). 26610004 10.1021/ct900086b

[b66] Best R. B. . Optimization of the additive CHARMM all-atom protein force field targeting improved sampling of the backbone *φ*, *ψ* and side-chain *χ*_1_ and *χ*_2_ dihedral angles. J. Chem. Theory Comput. 8, 3257–3273 (2012). 23341755 10.1021/ct300400x PMC3549273

[b67] Luo Y. & Roux B. Simulation of osmotic pressure in concentrated aqueous salt solutions. J. Phys. Chem. Lett. 1, 183–189 (2010).

[b68] Mark P. & Nilsson L. Structure and dynamics of the TIP3P, SPC, and SPC/E water models at 298 K. J. Phys. Chem. A 105, 9954–9960 (2001).

[b69] Humphrey W., Dalke A. & Schulten K. VMD: visual molecular dynamics. J. Mol. Graph. 14, 33–38 (1996). 8744570 10.1016/0263-7855(96)00018-5

