# Supplementary material for: Mechanism of pH-dependent activation of the sodium-proton antiporter NhaA
Source: Nat Commun. 2016 Oct 6;7:12940. doi: 10.1038/ncomms12940 (PMC5059715; doi:10.1038/ncomms12940)
Supplement: Supplementary Information — Supplementary Figures 1-20, Supplementary Tables 1-2, Supplementary Notes 1-2 and Supplementary References [file ncomms12940-s1.pdf]

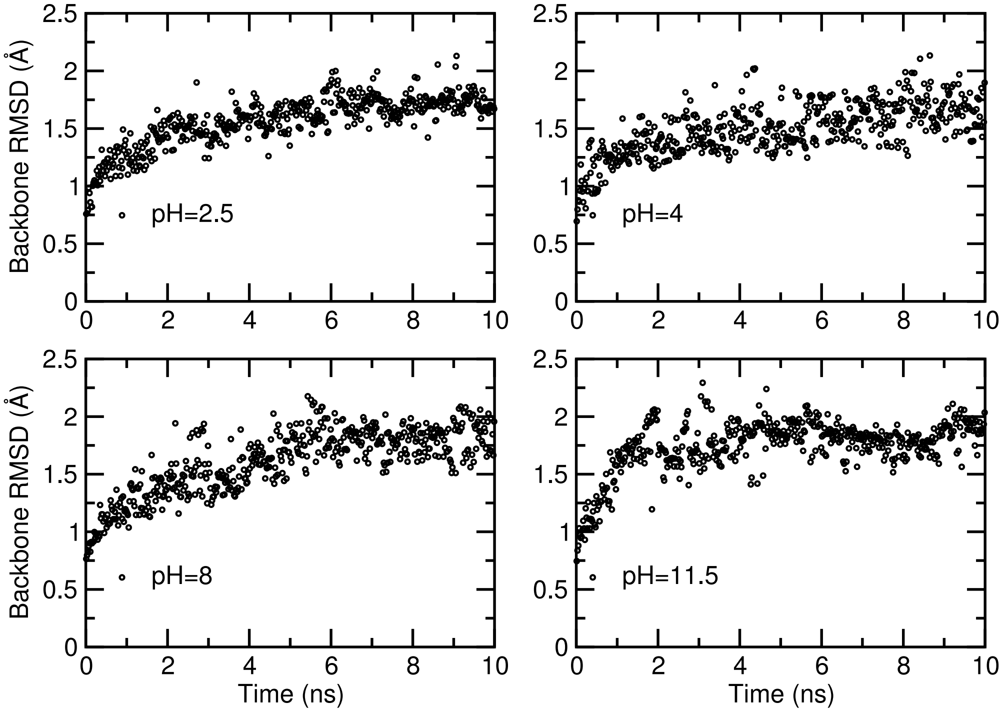

Supplementary Figure 1: Simulation convergence. Time series of backbone RMSD for simulation run 1 (4AU5).

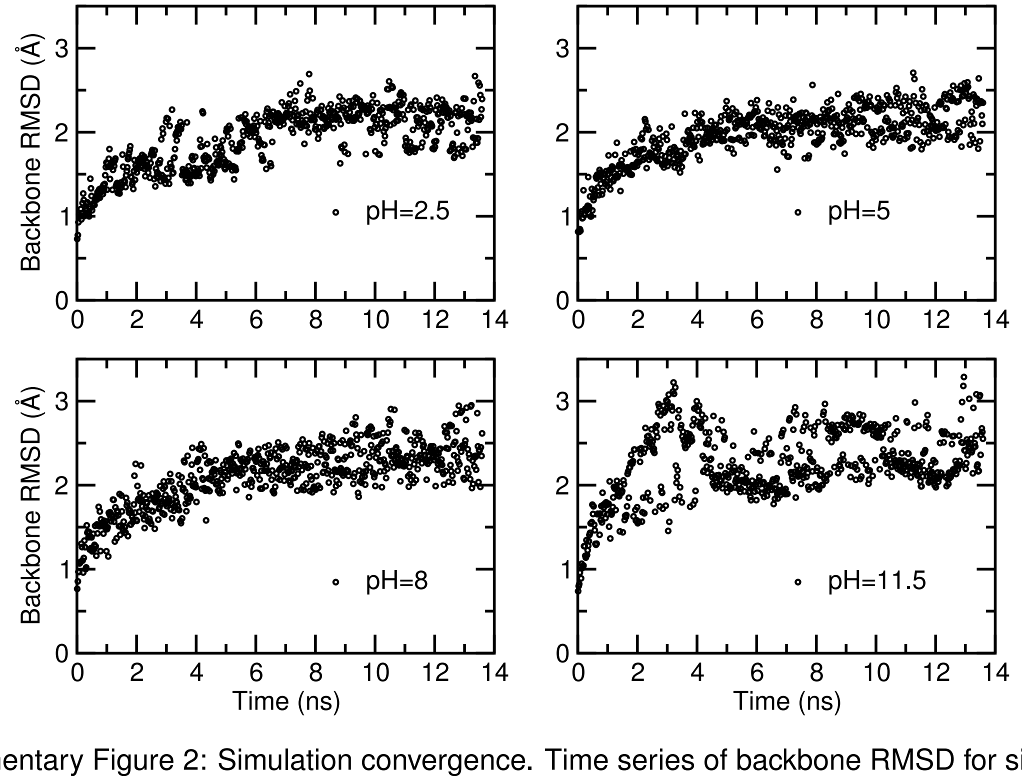

Supplementary Figure 2: Simulation convergence. Time series of backbone RMSD for simulation run 2 (1ZCD).

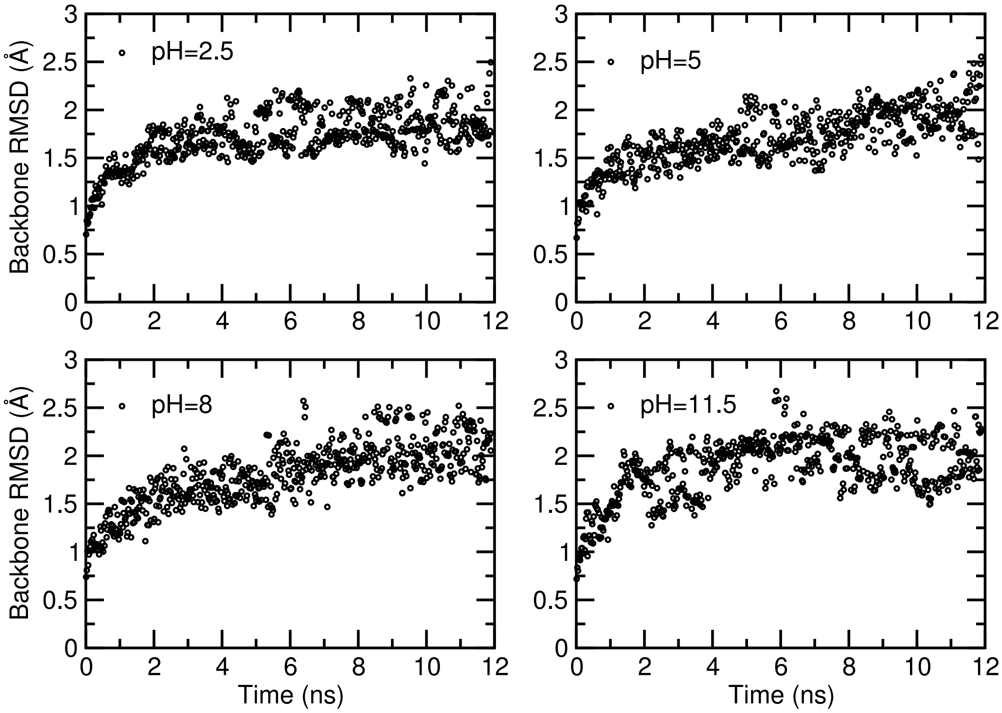

Supplementary Figure 3: Simulation convergence. Time series of backbone RMSD for simulation run 3 (1ZCD).

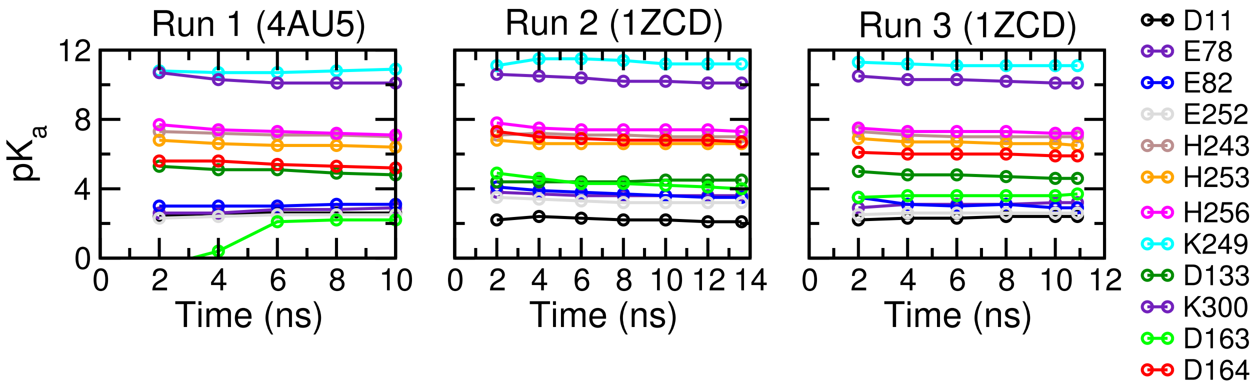

Supplementary Figure 4: Convergence of the calculated pK_a's. Cumulative pK_a values were calculated in 2-ns intervals. *Left*: simulation run 1 (initiated from the recent crystal structure with PDB ID: 4AU5). *Middle*: simulation run 2 (initiated from the previous crystal structure with PDB ID: 1ZCD). *Right*: simulation run 3 (initiated from the previous crystal structure with PDB ID: 1ZCD).

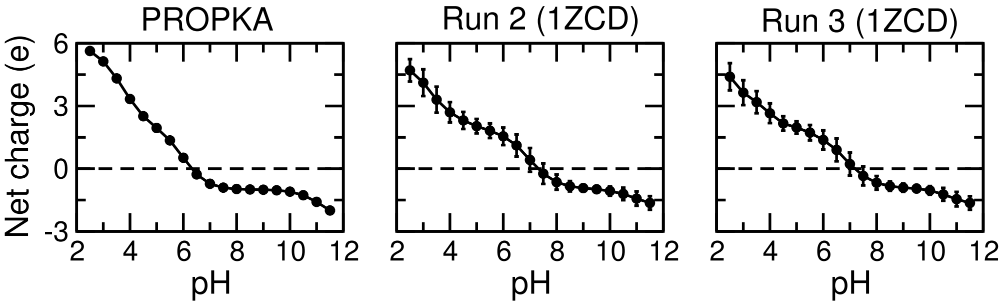

Supplementary Figure 5: Calculated total net charge of the pH sensor residues, Asp11, Glu78, Arg81, Glu82, His243, Lys249, Arg250, Glu252, His253, and His256. The error bars indicate the root-mean-squared fluctuations in the simulation. A horizontal line at net charge zero is drawn to guide the eye. Except for arginines which were kept in the charged state, all of the above residues were titrating in the simulation. The left panel presents data from the empirical PROPKA calculation [1] based on the snapshots from the conventional simulations.

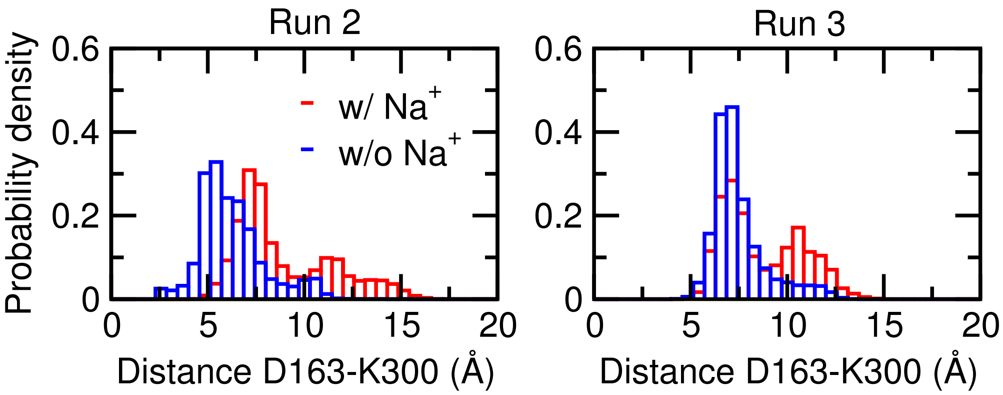

Supplementary Figure 6: Probability distribution of the minimum distance between the carboxylate oxygens of Asp163 and amine nitrogen of Lys300. Data in the absence and presence of sodium binding to Asp163 are shown in blue and magenta, respectively.

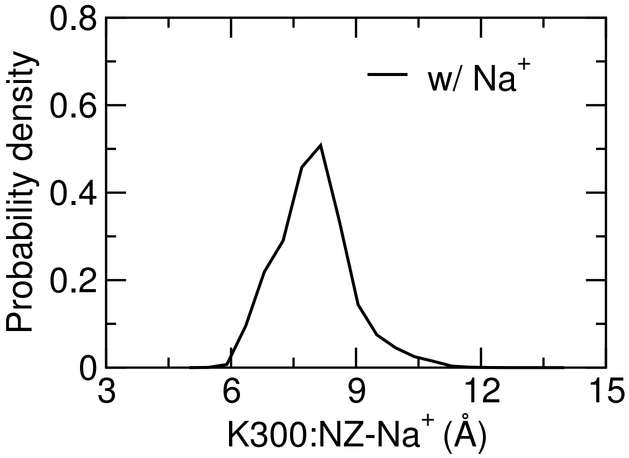

Supplementary Figure 7: Probability distribution of the distance between sodium and the amine nitrogen of Lys300 when sodium is bound to Asp163. Data from pH 9–11.5 of the CpHMD run 1 were used. These are the pH conditions with significant probability of sodium binding to Asp163 (see Fig. 5).

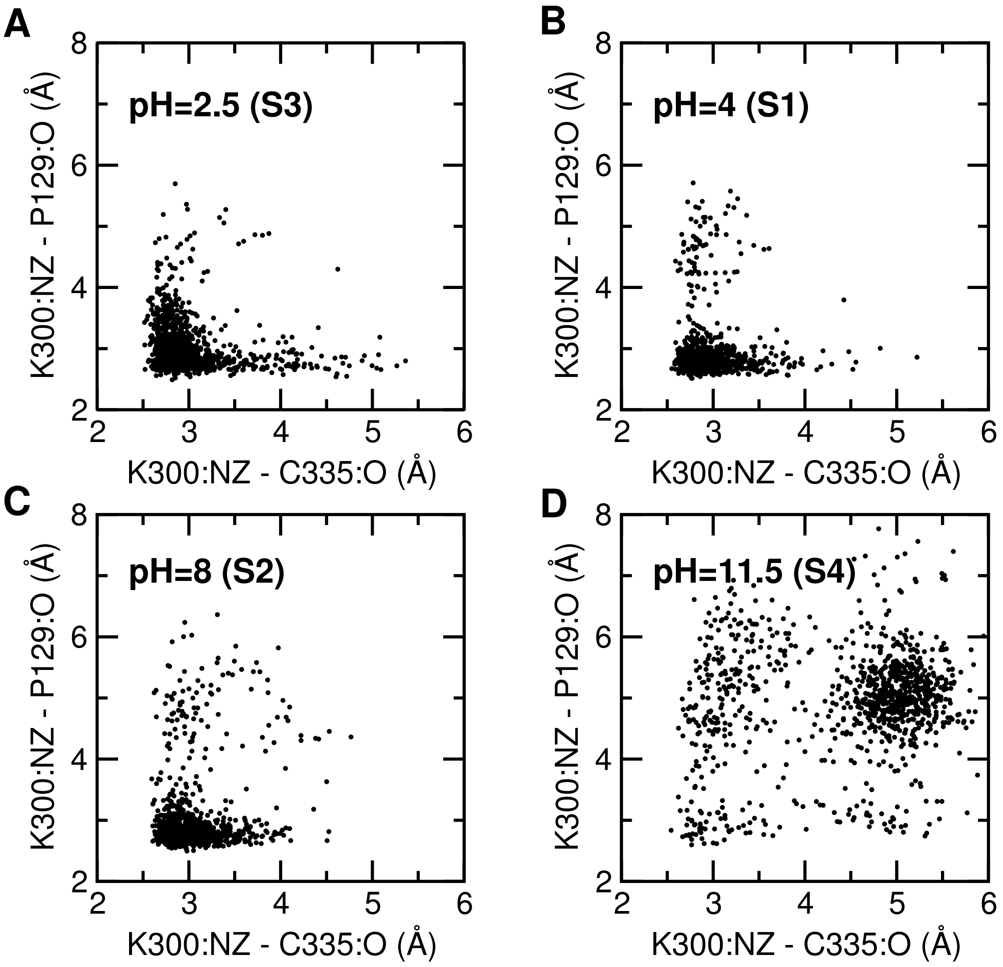

Supplementary Figure 8: The interactions between Lys300 and the negative dipoles of TM IVp and TM XIc are disrupted due to the neutralization of K300. Distance from Lys300 (amine nitrogen) to Cys335 on TM XIc (backbone carbonyl oxygen) vs. the distance to Pro129 on TM IVp (backbone carbonyl oxygen) at different pH conditions. The condition of charged Lys300 is represented by pH 2, 4 and 8 corresponding to the conventional simulations S3, S1 and S2, respectively. The condition of neutralized Lys300 is represented by pH 11.5. The CpHMD simulation run 1 was used.

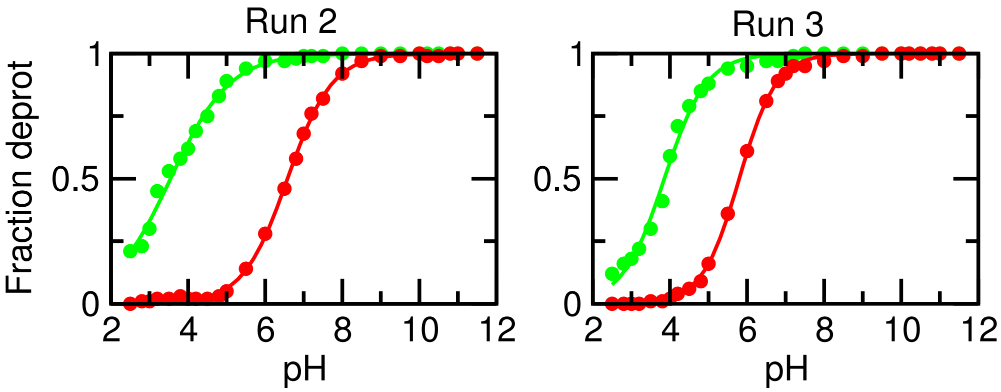

Supplementary Figure 9: Degree of deprotonation of Asp163 (cyan) and Asp164 (magenta) as a function of pH. Solid curve represents fitting to the generalized Henderson-Hasselbalch equation.

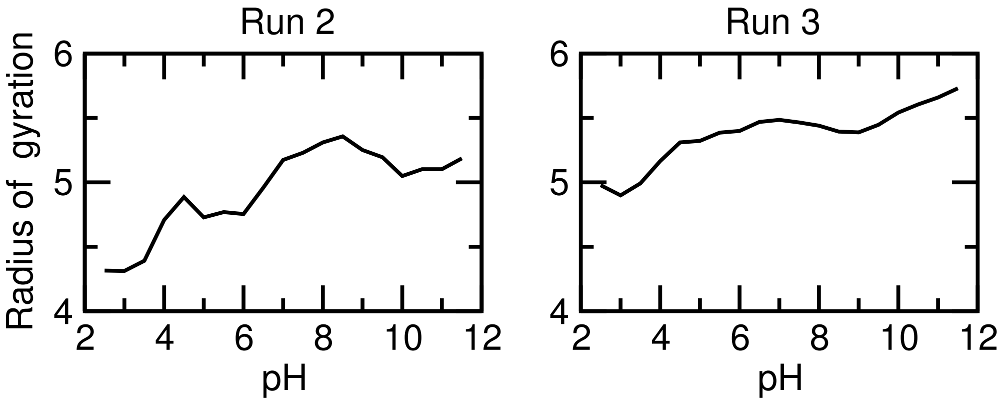

Supplementary Figure 10: Radius of gyration of the cytoplasmic gate (V75, I134, M157, A160 and I161) as a function of pH. Calculation was based on the sidechain heavy atoms. Each data point (for clarity not explicitly shown) is an average over all frames at a given pH.

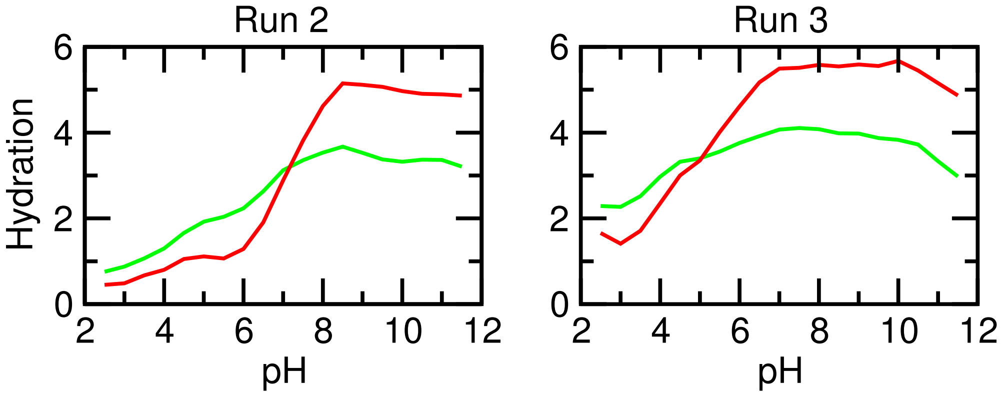

Supplementary Figure 11: Hydration number of Asp163 (cyan) and Asp164 (magenta) as a function of pH. Hydration number refers to the number of water in the first solvation shell, *i.e.*, any water oxygen within 3.5 Å from either of the carboxylate oxygens of Asp. Each data point (for clarity not explicitly shown) is an average over all frames at a given pH.

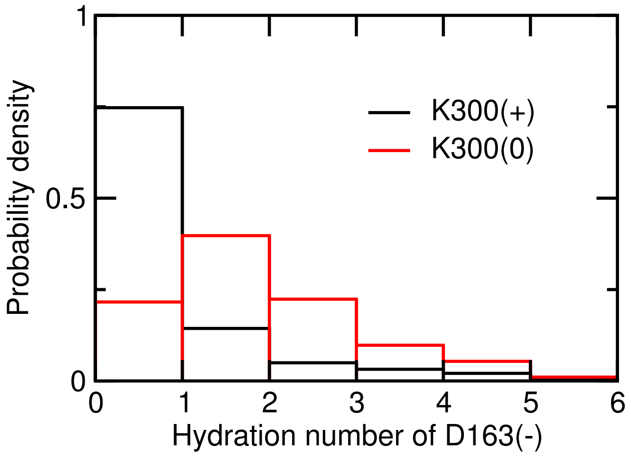

Supplementary Figure 12: Hydration of Asp163 increases as Lys300 becomes deprotonated. Histograms of the hydration number of deprotonated Asp163 when Lys300 is protonated (black) and deprotonated (magenta). The analysis is based on the CpHMD simulation run 1 using frames from the replicas at pH 5 and above.

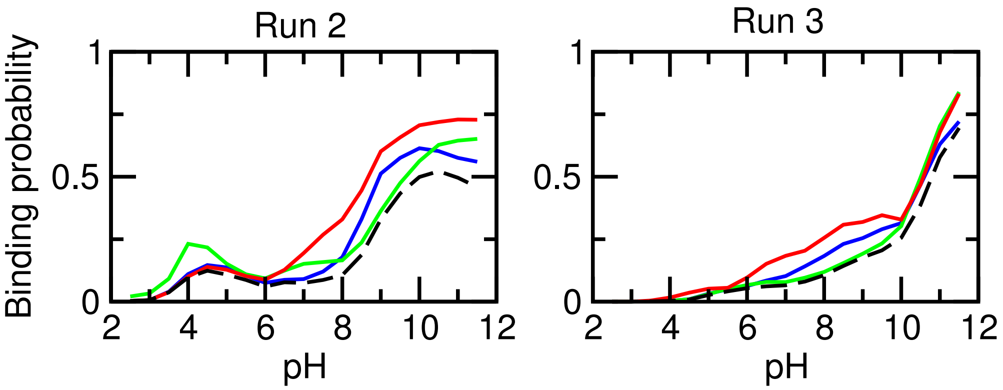

Supplementary Figure 13: Probability of sodium binding to Thr132 (blue), Asp163 (cyan) and Asp164 (magenta) as a function of pH. Black dashed curve represents the probability of simultaneous binding to all three residues.

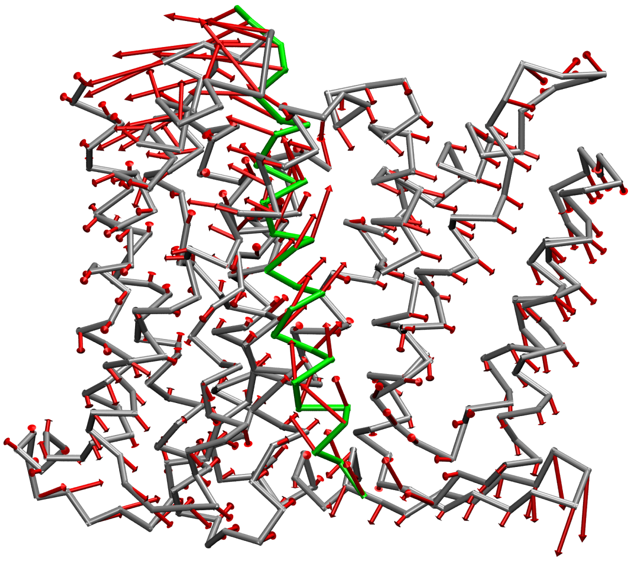

Supplementary Figure 14: Motion of the core relative to the dimerization domain. The first mode from the principal component analysis performed for simulation run 2 where bending of TM V was observed. Arrows represent the directions and magnitudes of motions of Cα atoms. TM V is colored green.

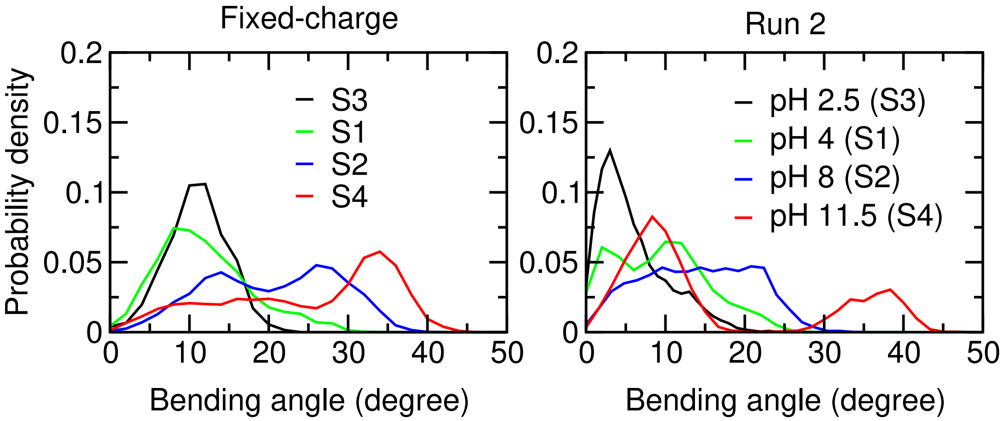

Supplementary Figure 15: Probability distribution of the TM V bending angle. **Left.** Data obtained from the conventional simulations initiated from the recent crystal structure (PDB ID 4AU5). **Right.** Data obtained from CpHMD simulation run 2 initiated from the previous crystal structure (PDB ID 1ZCD). The CpHMD simulations at pH 2.5, 4, 8, and 11.5 correspond to the conventional simulations S3, S1, S2 and S4 (see Table S1). S3: Asp163(0)/Asp164(0)/Lys300(+); S1: Asp163(-)/Asp164(0)/Lys(+); S2: Asp163(-)/Asp164(-)/Lys300(+); and S4: Asp163(-)/Asp164(-)/Lys300(0). Bending angle is defined as the deviation of the angle formed by the Cα atoms of Leu152, Asp163 and Phe174 on TM V from linearity. Bending angles in the previous (PDB ID 1ZCD) and current (PDB ID 4AU5) crystal structures are 4.9° and 5.7°, respectively.

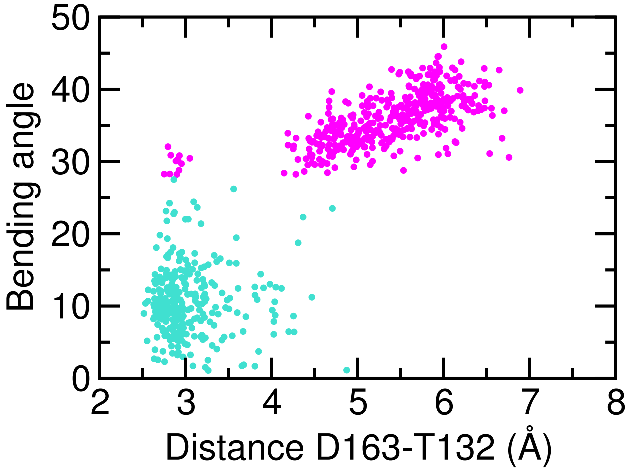

Supplementary Figure 16: Correlation between the TM V bending angle and the D163-T132 hydrogen bonding. Distance is measured between the carboxyl atoms of Asp163 and the amide nitrogen of Thr132. Data points with the bending angle smaller and larger than 28 degree are indicated in cyan and magenta data, respectively. Data obtained from CpHMD simulation run 2 initiated from the previous crystal structure (PDB ID 1ZCD).

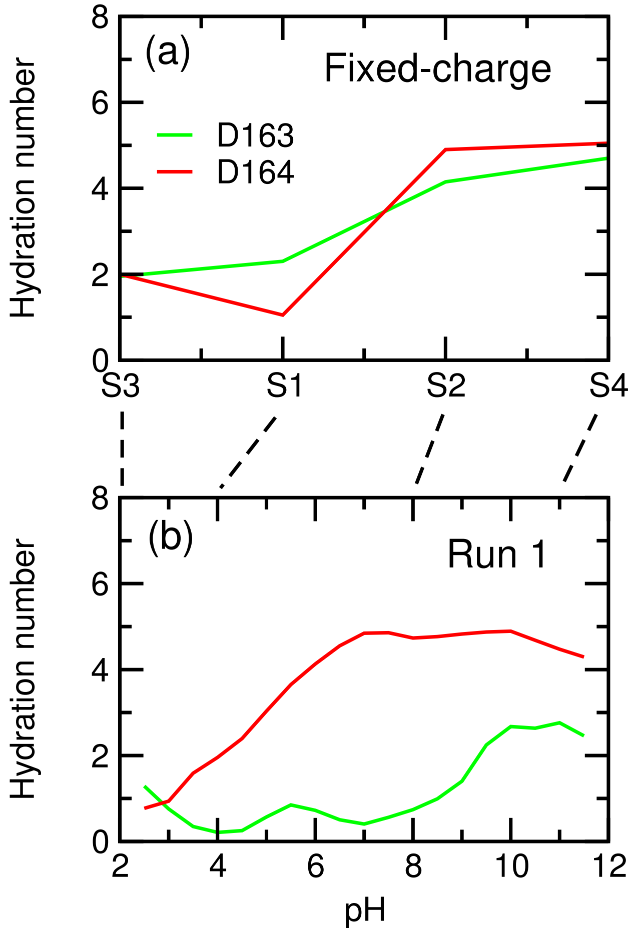

Supplementary Figure 17: Comparison of hydration numbers between conventional and CpHMD simulations. The average number of water molecules within 3.5 Å radius of the carboxylate moieties of the residues Asp163 (cyan) and Asp164 (magenta) is shown. (a) Conventional MD simulations. Names as in Table S1. (b) CpHMD simulations. Dashed lines very approximately indicate the correspondence between the configurations from the conventional MD and the pH conditions of the CpHMD simulations.

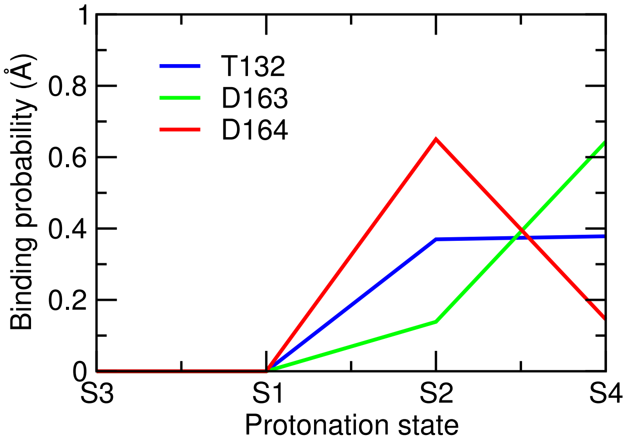

Supplementary Figure 18: Probability of sodium binding to Thr132 (blue), Asp163 (cyan) or Asp164 (magenta) for the four different fixed-charge simulations.

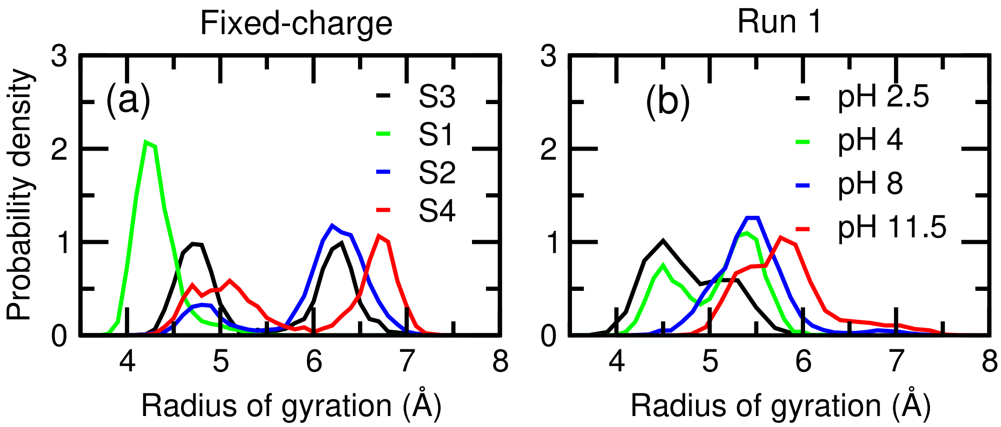

Supplementary Figure 19: Comparison of the opening size of the cytoplasmic hydrophobic gate between the conventional (a) and CpHMD (b) simulations. The size of the gate is quantified by the radius of gyration of the hydrophobic residues Val75, Ile134, Met157, Ala160, and Ile161. pH windows in (b) roughly correspond to the fixed charge simulations in (a). The gate radii for the two NhaA crystal structures with PDB ID 1ZCD and 4AU5 are 4.2 and 4.5 Å respectively.

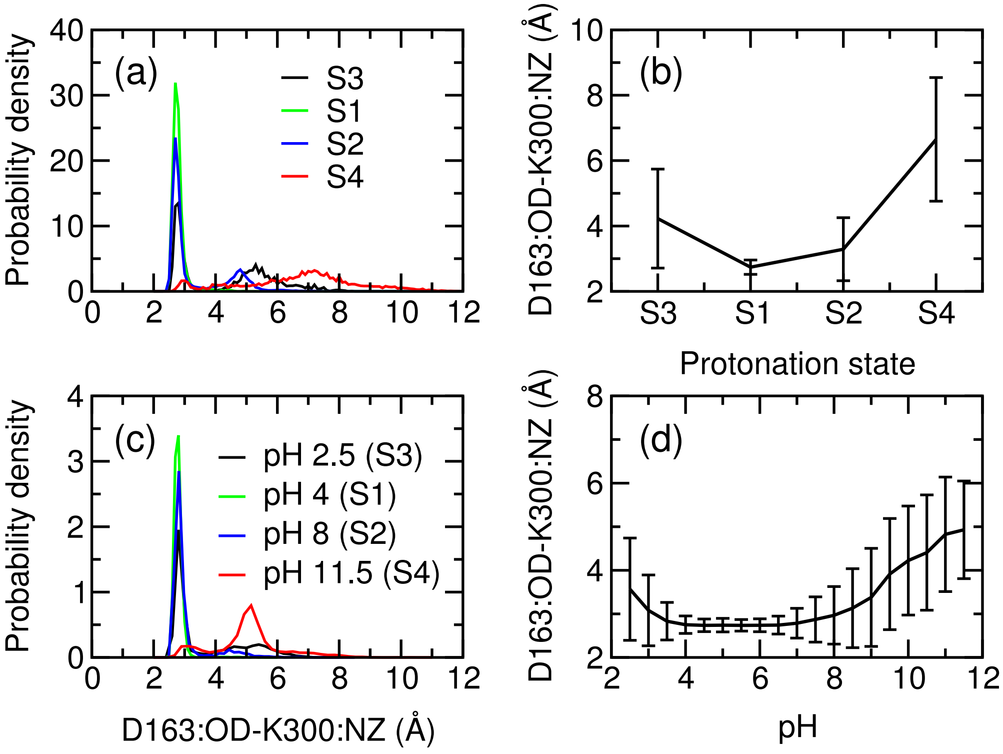

Supplementary Figure 20: Comparison of the Asp163–Lys300 interaction in the conventional and CpHMD simulations. (a) Distribution of the Asp163–Lys300 distance from the conventional simulations S1 – S4 (see Table S1). (b) Average Asp163–Lys300 distance from the conventional simulations. Error bars indicate one standard deviation of the data. (c) Distribution of the Asp163–Lys300 distance from CpHMD simulation run 1. Selected pH conditions roughly correspond to the conventional simulations S1–S4. (d) Average Asp163–Lys300 distance from CpHMD simulation run 1. Error bars indicate one standard deviation of the data. The Asp163–Lys300 distances in the two NhaA crystal structures with PDB ID 1ZCD and 4AU5 are 11.74 and 2.55 Å, respectively.

Supplementary Table 1: Summary of CpHMD and conventional MD simulations

**CpHMD**				
	PDB	pH range	No. replicas	Length (ns)
Run 1	4AU5	1.5–11.5	28	280.0 (10.0/replica)
Run 2	1ZCD	2.5–11.5	28	380.8 (13.6/replica)
Run 3	1ZCD	2.5–11.5	28	336.0 (12.0/replica)
**Conventional MD with POPC**[a]				
	PDB	Charge state		Length (ns)
S3	4AU5	D163(0)/D164(0)/K300(+)		1000
S1	4AU5	D163(-)/D164(0)/K300(+)		1400
S2	4AU5	D163(-)/D164(-)/K300(+)		3000
S4	4AU5	D163(-)/D164(-)/K300(0)		2200
**Conventional MD with 4:1POPE:POPG**[b]				
	PDB	Charge state		Length (ns)
S1	4AU5	D163(-)/D164(0)/K300(+)		30
S2	4AU5	D163(-)/D164(-)/K300(+)		470.5
S4	4AU5	D163(-)/D164(-)/K300(0)		30

[a]For simulation details see ref [2]. [b]These simulations were performed to verify if there is lipid dependence. It was found that sodium binding occurs in S2 and S4 but not in S1. Further, the binding sites are T132/D163/D164. Thus, the conclusions in the main text remain the same regardless whether POPC or a 4:1 POPE:POPG mixed membrane (which approximates the native E. coli inner membrane) is used, consistent with a recent review which found that, unlike ion channels, evidence of lipid dependence for transporters is scant [3].

Supplementary Table 2: Calculated pK_a's of NhaA from three CpHMD runs

Residue	run 1	run 2	run 3
**pH Sensor**			
Asp11	2.6	$<$2.5	$<$2.5
Glu78	3.1	3.7	3.6
Glu82	3.3	3.2	2.6
His243	6.8	6.9	7.0
Lys249	11.2	11.1	11.0
Glu252	2.6	3.1	2.7
His253	6.3	6.5	6.3
His256	6.9	7.2	7.0
**Core**			
Asp133	4.5	4.7	4.4
Asp163	2.4	3.5	3.9
Asp164	5.0	6.6	5.8
Lys300	10.1	10.0	10.0
**Other**			
His39	7.7	8.0	7.8
Asp40	3.8	3.9	3.6
Glu43	3.7	3.9	4.0
Glu54	3.5	3.6	3.7
Lys57	11.1	$>$11.5	10.7
Asp65	4.9	4.6	4.3
Lys80	6.5	5.9	6.5
Asp119	3.1	3.0	3.3
Glu124	7.1	5.3	5.7
Lys153	10.0	10.4	10.6
Asp178	3.1	3.0	3.4
Lys221	9.7	10.1	9.8
His225	$<$2.5	$<$2.5	$<$2.5
Lys240	10.3	10.3	10.4
Glu241	3.5	3.4	2.9
Lys242	10.4	10.2	10.2
Asp282	4.1	3.9	4.2
Lys315	10.5	9.6	10.0
His318	7.1	7.0	7.4
Glu321	4.2	4.0	3.8
Asp354	4.6	3.4	4.0
Glu356	4.0	4.1	4.2
Lys362	10.3	10.4	10.6

The model pK_a values for Asp, Glu, His and Lys are 4.0, 4.4, 6.45 and 10.4, respectively [4]. The pK_a of His225 could not be accurately calculated, as it extends into the bilayer and the membrane-GBSW model overestimates the desolvation free energy in such an environment, i.e. pK_a of His225 is too low.

Supplementary Note 1 - Comparison to semi-macroscopic pK_a calculations

Below we compare the calculated pK_a's of K300 and D163 in Ref. [5] and our results based on the same crystal structure (PDB ID 4AU5). In Ref. [5] the calculated pK_a of K300 was 12.81, which is 2.81 units higher than our calculated value of 10.1 considering all configurations, with and without sodium binding to D163. However, if separating the sodium-bound and unbound configurations, the CpHMD predicted pK_a's are 8.9 and 11.6, respectively. The latter is higher than the model value of 10.4, in agreement with the value in Ref. [5](12.81). This is within expectation, since in the pK_a calculation of Ref. [5], sodium binding was not taken into account (according to our reading of the paper).

Now we compare the calculated pK_a's of D163. In Ref. [5], the calculation using implicit charge (assuming neutral background) gave 7.28 with D164(-) and 4.66 with D164(0); the calculation assuming D133(-1)/K300(+) gave 8.4 with D164(-) and 6.5 with D164(0). In our work, the calculated pK_a of D163 was 2.4. This value is not affected by sodium binding, because the latter occurs at a much higher pH (above 6) than the titration range of D163. Since our calculated pK_a's of D133 and D164 were 4.5 and 5.0, both groups were neutral in the titration range of D163. Thus, our calculated pK_a of D163 really corresponds to the result in Ref. [5] considering D164(0) and K300(+). Given that Ref. [5] gave 4.66 for the pK_a of D163 with D164(0)/K300(0), we anticipate the pK_a of D163 with D164(0)/K300(+) to shift lower (due to electrostatic attraction of K300), bringing it closer to our value of 2.4. Thus, we think the microscopic pK_a's of K300 and D163 obtained by us and in Ref. [5] are in qualitative agreement.

Supplementary Note 2 - Agreement between CpHMD, conventional fixed-protonation-state simulations and new crystal structure

A major limitation of the current CpHMD implementation is the relatively short time scale (about ten nanoseconds per pH replica), which may result in the incomplete sampling of conformational states of protein and solvent despite the use of the pH replica-exchange enhanced sampling protocol. The conventional fixed-protonation-state simulations discussed here, on the other hand, were repeated for different combinations of protonation states up to three times for the total sampling time between 1 μs and 3 μs (Supplementary Table 1), thus providing perhaps more complete conformational sampling. In order to establish the degree of convergence of the CpHMD simulations we compare key quantities with the conventional simulations. The hydration levels for Asp133, Asp163, and Asp164 in each conventional simulation match the hydration levels in the CpHMD simulations at the corresponding pH conditions. Specifically, the conventional simulations with Asp163/Asp164/Lys300 protonated (named S3) correspond to about pH 2; the simulations with Asp163 deprotonated and Asp164/Lys300 protonated (S1) to pH 4; Asp163/Asp164 deprotonated and Lys300 protonated (S2) to pH 8; and Asp163/Asp164/Lys300 deprotonated (S4) to pH 11.5 (Supplementary Fig. 15). The correspondence over the whole pH range is remarkable, because the two sets of simulations used different force fields and water models (CHARMM with TIP3P for CpHMD, OPLS-AA with TIP4P for the conventional MD). Thus, hydration levels appear to be converged in the CpHMD simulations and can serve as a robust quantity to compare the two simulation approaches with each other and to assign an effective pH to the conventional simulations.

The probability of sodium binding to Asp163, Asp164 and Thr132 is also similar when comparing conventional MD data (Supplementary Fig. 16) with the corresponding pH windows in CpHMD (Fig. 5 in main text). The CpHMD simulations are clearly capable of capturing this key event in the proposed model. The opening of the cytoplasmic hydrophobic gate across the pH range (Fig. 4 in main text) is also qualitatively recapitulated in the conventional MD (Supplementary Fig. 17), with the long simulations showing larger openings than the shorter CpHMD. It is possible that longer time-scale rearrangements in the gate region are not fully captured in the CpHMD simulations but the gate is already seen to open sufficiently to provide water molecules and sodium ions full access to the binding site.

The Asp163–Lys300 salt bridge shows very similar behavior in the conventional MD and CpHMD simulations (Supplementary Fig. 18). At high pH ($>$8) the salt bridge is predominantly broken because a sodium ion can bind to Asp163 and compete with the salt bridge and thus further stabilize the deprotonated (neutral) charge state of Lys300. At very low pH ($<$3), the salt bridge is also weakened and often found dissociated because Asp163 is protonated and therefore the electrostatic interaction to Lys300 is weakened. In the intermediate pH range, both Asp163 and Lys300 are charged and the strong Coulomb interaction favors the salt bridge with a well-defined distance of about 2.8 Å. The new NhaA crystal structure was solved at pH 3.5 [2] and contains the salt bridge, as also seen in the S1 and S2 simulations. The CpHMD simulations now clearly explain this observation as a consequence of the very low pK_a of Asp163. The consistency between low-pH crystal structure and both sets of simulations also corroborates the hypothesis that Asp163 is not one of the proton carriers because it remains charged at all physiologically accessible pH conditions.

Supplementary References

[1] Søndergaard, C. R., Mats H. M. Olsson, M. R. & Jensen, J. H. Improved Treatment of Ligands and Coupling Effects in Empirical Calculation and Rationalization of pKa Values. *J. Chem. Theory Comput.* **7**, 2284–2295 (2011).

[2] Lee, C. *et al.* Crystal structure of the sodium-proton antiporter NhaA dimer and new mechanistic insights. *J. Gen. Physiol.* **144**, 529–544 (2014).

[3] Denning, E. J. & Beckstein, O. Influence of lipids on protein-mediated transmembrane transport. *Chem. Phys. Lipids* **169**, 57–71 (2013).

[4] Nozaki, Y. & Tanford, C. Examination of titration behavior. *Methods Enzymol.* **11**, 715–734 (1967).

[5] Alhadeff, R. & Warshel, A. Simulating the function of sodium/proton antiporters. *Proc. Natl. Acad. Sci. USA* **112**, 12378–12383 (2015).
